# Red OLED with efficiency of 25.6% at 10,000 cd m^−2^ based on selenium embedding multiple resonance framework

**DOI:** 10.1038/s41377-026-02220-w

**Published:** 2026-04-08

**Authors:** Yexuan Pu, Xinliang Cai, Chenglong Li, Baoyan Liang, Hai Bi, Yue Wang

**Affiliations:** 1 State Key Laboratory of Supramolecular Structure and Materials, College of Chemistry, Jilin University, Changchun, 130012 China; 2 Jihua Laboratory, 28 Huandao South Road, Foshan, 528200 Guangdong Province China

**Keywords:** Organic LEDs, Photonic devices

## Abstract

Multiple resonance thermally activated delayed fluorescence (MR-TADF) materials featuring narrowband emission and high luminescence efficiency hold great promise for ultra-high-definition displays. However, red MR emitter-based organic light-emitting diodes (OLEDs) commonly suffer from pronounced efficiency roll-off due to intrinsically slow reverse intersystem crossing (RISC), which severely hinders their practical application. Herein, we present efficient OLEDs based on a selenium-embedded red MR framework featuring fast RISC, which not only serves as a high-performance emitter but also functions as a sensitizer. The emitter (tFSeBN) shows red emission at 607 nm and achieves a record-high RISC rate of 7.5 × 10^5^ s^–^^1^. The corresponding OLED delivers a maximum external quantum efficiency (EQE_max_) of 34.7% and maintains high EQE values of 31.0% and 25.6% at luminance levels of 1000 and 10,000 cd m^−2^, highlighting its ultra-low efficiency roll-off. Owing to its high tolerance to doping concentration and accelerated RISC, tFSeBN further serves as an efficient sensitizer in hyperfluorescent OLEDs, enabling pure-red emission with CIE coordinates of (0.70, 0.30), high EQE and suppressed efficiency roll-off. This work provides a viable pathway to address the long-standing efficiency roll-off issue in red MR-OLEDs, serving as an alternative to conventional noble-metal-sensitized architectures for the red OLED industry.

## Abstract (web summary)

A red multiple-resonance molecule with fast reverse intersystem crossing functions as both emitter and sensitizer, enabling efficient Organic Light-Emitting Diodes with suppressed efficiency roll-off.

## Graphical abstract

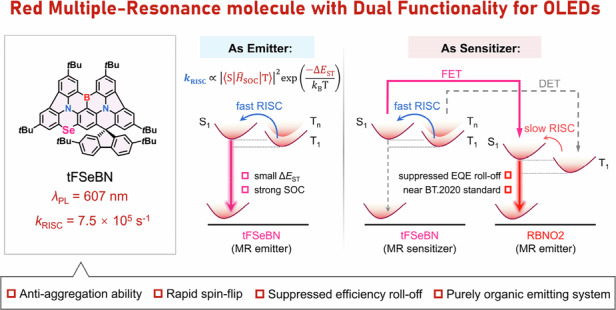

## Introduction

Owing to their advantages of self-emissive nature, high contrast, fast response times, and mechanical flexibility, organic light-emitting diodes (OLEDs) have emerged as a key technology in modern high-end displays^1–3^. In pursuit of superior visual experience, OLED technology is rapidly advancing towards wider color gamut and better brightness performance^4^. Among various OLEDs, the multiple resonance thermally activated delayed fluorescence (MR-TADF) based OLEDs have attracted considerable attention for their outstanding color purity and high efficiency^5–10^. For MR-TADF emitters, the complementary resonance effects of boron and nitrogen atoms induce the alternate localization of frontier molecular orbitals (FMOs), which suppresses the structural relaxation and vibronic coupling, resulting in high photoluminescence quantum yield (*Ф*_PL_) and narrowband emission. However, a critical challenge for MR-TADF materials is their intrinsically slow reverse intersystem crossing (RISC) process^11–13^. When operating at high luminance, the rapid accumulation of triplet excitons cannot be efficiently and timely upconverted due to the limited RISC rate (*k*_RISC_), thereby increasing exciton losses through nonradiative pathways associated with triplet–triplet annihilation (TTA) and singlet–triplet annihilation (STA). Most reported MR-TADF OLEDs show severe efficiency roll-off at luminance above 1000 cd m^–^^2^, far below practical display requirements.

**Fig. 1:**
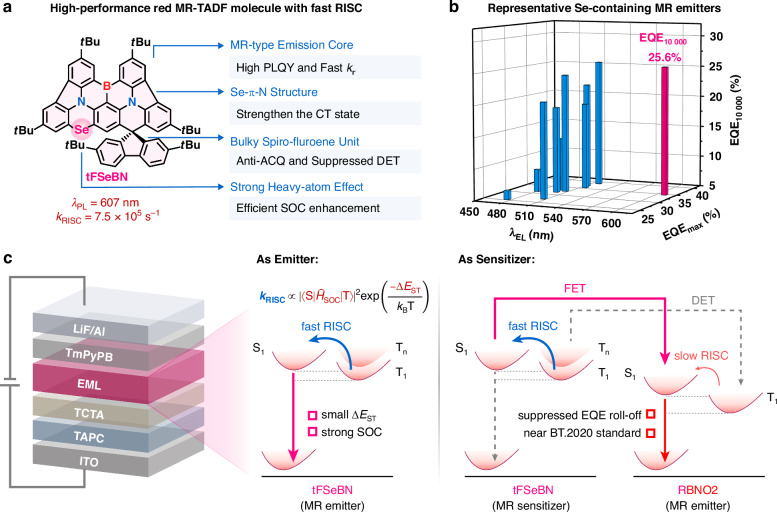
Design strategy. **a** Molecular structure of tFSeBN. **b** Electroluminescence performance of representative Se-containing MR-TADF emitters. (pink indicates tFSeBN; blue indicates other reported molecules) **c** The energy pathways of employing tFSeBN as the emitter and sensitizer, respectively

While various strategies have been successfully developed to suppress the efficiency roll-off in blue and green MR-TADF OLEDs^14–21^, progress in the red region has critically lagged behind^22–24^. At present, the widely adopted approach to mitigate severe roll-off in red MR-TADF devices is the sensitization architecture, where the electrogenerated excitons can be efficiently harvested by a sensitizer and subsequently transferred to the terminal emitter via the Förster energy transfer (FET) process. However, sensitizers that simultaneously satisfy spectral-matching and triplet-management requirements for red MR emitters remain extremely limited. To date, noble-metal-based phosphorescent sensitizers have been virtually indispensable in the fabrication of high-performance red MR-TADF OLEDs^25–29^. This reliance faces challenges of resource scarcity and high device manufacturing costs, severely limiting the advancement of the OLED industry. Therefore, developing red MR-TADF emitters with intrinsically fast *k*_RISC_ and expanding sensitizer options beyond noble-metal complexes have become urgent and important tasks toward practical OLED applications. In this contribution, we present a selenium (Se)-atom embedded MR framework featuring intrinsically fast *k*_RISC_ (Fig. 1a). The introduction of an electron-donating atom guarantees a reduction in the singlet–triplet energy gap (Δ*E*_ST_) and red-shifted emission. Meanwhile, the Se atom (Z_*N*_ = 34) possesses a strong heavy-atom effect, and its large atomic radius promotes molecular structural distortion, which is an important structural foundation for effective spin–orbit coupling (SOC) enhancement. This synergistic modulation of electronic and geometric factors through a single-atom engineering promotes *k*_RISC_ to reach a high level, paving a viable pathway toward red MR OLEDs with intrinsically suppressed efficiency roll-off.

The parent MR framework tFBN was constructed by integrating the classical MR core (DtBuCzB)^30^ with a bulky tert-butyl-substituted fluorene unit to suppress aggregation-caused quenching (ACQ). The Se-containing emitter tFSeBN was synthesized via a one-shot process from tFBN, exhibiting a red emission peaking at 607 nm in toluene, with a full width at half maximum (FWHM) of 48 nm (0.16 eV) and a high *Ф*_PL_ of 98%. Meanwhile, its sulfur (S)-containing analog tFSBN was also developed for comparison and to gain insights into structure-property relationships. As envisioned, the introduction of S raises the *k*_RISC_ value from 9.0 × 10^3^ s^–^^1^ (tFBN) to 3.2 × 10^4 ^s^–^^1^ (tFSBN), while Se leads to a boosted *k*_RISC_ of 7.5 × 10^5^ s^–^^1^ (tFSeBN). The non-sensitized OLED based on tFSeBN delivers an outstanding maximum external quantum efficiency (EQE_max_) of 34.7%, along with ultra-low efficiency roll-off, maintaining EQEs of 31.0% and 25.6% at elevated luminance levels of 1000 and 10,000 cd m^−2^, respectively. Notably, the exceptional EQE of up to 25.6% under 10,000 cd m^–^^2^ marks a significant advance for red MR-TADF devices and even ranks among the highest efficiencies reported to date for Se-containing MR-TADF devices (Fig. 1b and Table S1). Moreover, its synergistic advantages in fast RISC and high tolerance to doping concentration render tFSeBN not only a high-performance red MR emitter, but also an efficient sensitizer for hyperfluorescence (HF) OLEDs. When tFSeBN is employed to sensitize the reported red emitter RBNO2^25^, efficient energy transformation from tFSeBN to RBNO2 is achieved. The resulting HF device achieves a pure red emission with Commission Internationale de l’Éclairage (CIE) coordinates of (0.70, 0.30), an improved EQE_max_ of 28.5%, and remarkably suppressed efficiency roll-off at high brightness. This work provides an effective approach to tackle the efficiency roll-off concern in red MR-TADF devices and may offer a viable strategy for advancing noble-metal-free OLED technologies.

## Results

The synthetic routes for the three MR-TADF compounds are illustrated in Scheme S1, and detailed procedures are provided in the Supporting Information. Under iodine catalysis, direct incorporation of S or Se into the highest occupied molecular orbital (HOMO) positions of tFBN afforded the target compounds tFSBN and tFSeBN, respectively, both in high yields. Their chemical structures were verified through comprehensive spectroscopic analysis (Figs. S1–S9). According to the cyclic voltammetry (CV) measurement (Fig. S10), the HOMO and the lowest unoccupied molecular orbital (LUMO) energy levels are −5.17 eV and −2.57 eV for tFBN, −4.83 eV and −2.68 eV for tFSBN, and −4.84 eV and −2.67 eV for tFSeBN. Thermogravimetric analysis (TGA) demonstrates that these three molecules exhibit high decomposition temperatures (*T*_d_, defined as 5% weight loss) exceeding 400 °C (Fig. S11), indicating their desirable thermal stability suitable for the vacuum sublimation purification and thermal evaporation OLED fabrication.

**Fig. 2:**
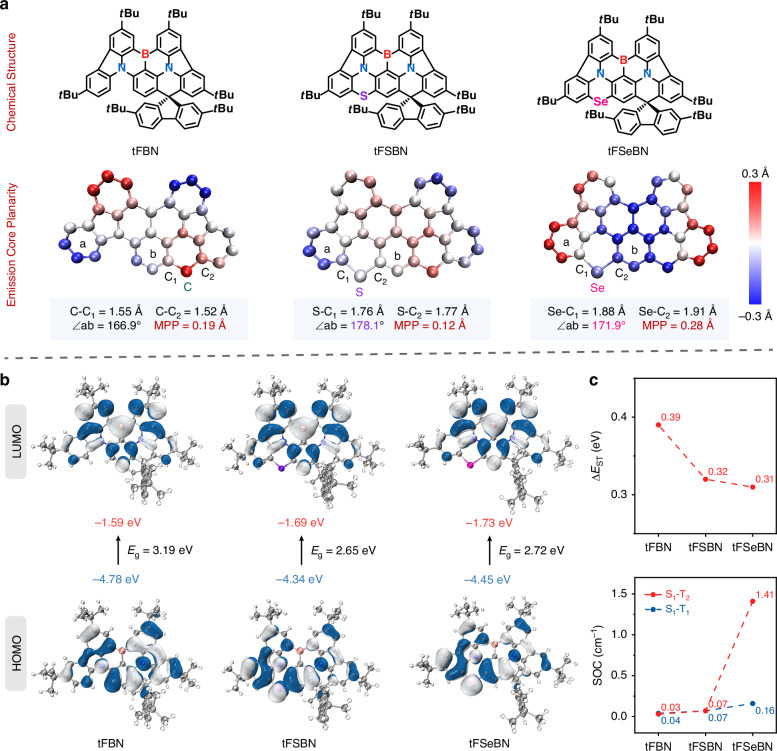
Theoretical analysis. **a** Chemical structures of tFBN, tFSBN and tFSeBN and calculated molecular planarity parameters (MPPs) (darker colors indicating a greater deviation from the fitted plane). **b** The HOMO and LUMO distributions; **c** the energy gaps (Δ*E*_ST_) between the first singlet and triplet states and spin–orbit coupling (SOC) matrix elements of tFBN, tFSBN and tFSeBN

X-ray crystallographic analysis was conducted to investigate the molecular geometries of the emitters. While single crystals of tFBN and tFSBN were successfully obtained, attempts to grow crystals of tFSeBN were unsuccessful. As an alternative, we employed FSeBN, a simplified analog of tFSeBN lacking two tert-butyl groups to facilitate crystallization (Fig. S12 and Tables S2–S4). The UV–vis absorption and photoluminescence (PL) spectra of FSeBN are nearly identical to those of tFSeBN (Fig. S13), confirming that removal of the tert-butyl groups does not significantly perturb the electronic structure. Therefore, FSeBN can serve as a reliable model for analyzing the geometric features of the Se-containing MR core. As the atomic number increases from C to S and to Se, the bond lengths between them and the carbon atom gradually increase (C–C: 1.52–1.55 Å; S–C: 1.76–1.77 Å; Se–C: 1.88–1.91 Å) (Fig. 2a). The dihedral angles between the two aryl planes (denoted as a and b) connected to the heavy atoms decrease from 178.1° in tFSBN to 171.9° in FSeBN. Therefore, the Se-containing framework shows the largest molecular planarity parameter (MPP = 0.28 Å), confirming its more distorted geometry^31^.

To gain deeper insight into the impact of heavy-atom embedding on the molecular optoelectronic properties, we performed density functional theory (DFT) and time-dependent DFT (TD-DFT) calculations at the B3LYP/6-31 G (d, p) level (Fig. 2b, c). The FMOs of all three emitters display spatial separation patterns with the MR characteristics, which are essential for achieving narrowband emission. The incorporation of an electron-donating S or Se atom elevates the HOMO energy levels and stabilizes the LUMO energy levels, thus reducing the energy gap (*E*_g_) and causing a significant red-shift of the emissions. Notably, the more distorted geometry of tFSeBN weakens the p–π conjugation between the Se atom and the π-framework, leading to a slightly larger *E*_g_ (2.72 eV) compared to tFSBN (2.65 eV). As shown in Fig. 2c and Table S5, despite both tFSBN and tFSeBN exhibiting similarly reduced Δ*E*_ST_ (0.32 and 0.31 eV, respectively), their SOC values differ significantly. The heavy-atom effect of S in tFSBN is quite limited, as reflected by the SOC matrix elements (〈S_1_ | *Ĥ*_SOC_ | T_1_〉 = 0.07 cm^−1^, 〈S_1_ | *Ĥ*_SOC_ | T_2_〉 = 0.07 cm^−1^), which show a negligible increase compared with those of tFBN (〈S_1_ | *Ĥ*_SOC_ | T_1_〉 = 0.03 cm^−1^, 〈S_1_ | *Ĥ*_SOC_ | T_2_〉 = 0.04 cm^−1^). In contrast, tFSeBN demonstrates substantial SOC enhancement (〈S_1_ | *Ĥ*_SOC_ | T_1_〉 = 0.16 cm^−1^, 〈S_1_ | *Ĥ*_SOC_ | T_2_〉 = 1.41 cm^−1^). The folded geometry of tFSeBN provides an essential structural basis that allows the strong heavy-atom effect of Se to be effectively manifested, thereby enhancing SOC, as further supported by theoretical analysis in the Supplementary Information (Figs. S14–S15). Consequently, the synergistic effects of Δ*E*_ST_ reduction and SOC enhancement can substantially accelerate the RISC process in tFSeBN, in accordance with El-Sayed’s rule^32,33^. To further discuss the effects of heavy-atom incorporation on the vibrational characteristics of the MR framework, we calculated the reorganization energies (*λ*) and identified several dominant vibrational modes (Fig. S16). It is observed that the high-frequency modes (>500 cm^−1^) are primarily attributed to the stretching vibrations of C–C bonds within the molecular skeletons. tFSeBN with the heavier Se atom exhibits reduced reorganization energy (*λ*) contributions from high-frequency stretching vibrations compared to tFSBN. Therefore, the weakened vibrational coupling associated with the S_1_–S_0_ transition contributes to the narrower emission spectrum of tFSeBN.

**Fig. 3:**
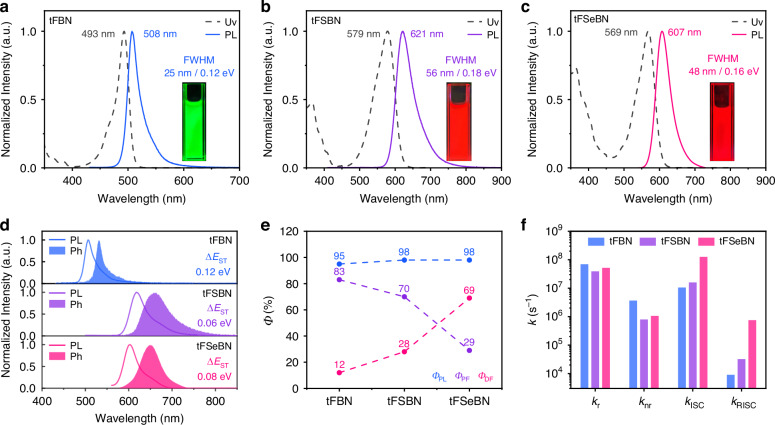
Photophysical properties. UV–vis absorption and fluorescence spectra of **a** tFBN, **b** tFSBN, and **c** tFSeBN in toluene solution (1 × 10^−5^ M, 298 K) (insert: photograph taken under 365 nm UV light). **d** Fluorescence and phosphorescence spectra tFBN, tFSBN, and tFSeBN in toluene solution (1 × 10^−5^ M, 77 K). **e** Comparison of the *Φ*_PL_ values and **f** the calculated rate constants for thin films of 1 wt% tFBN, tFSBN, and tFSeBN doped in the DMIC-TRZ host

The photophysical properties of tFBN, tFSBN and tFSeBN were investigated in dilute toluene solution (Fig. 3a–d and Table 1). All three emitters exhibit intense short-range charge transfer (SRCT) absorption bands in the ultraviolet–visible (UV–vis) spectra. Derived from the absorption onset, the optical energy gaps decrease from 2.44 eV (tFBN) to 2.01 eV (tFSBN) and 2.05 eV (tFSeBN), consistent with the computational results. Under 365 nm ultraviolet illumination, tFBN exhibits green fluorescence (508 nm), while tFSBN and tFSeBN show bright red emission peaking at 621 nm and 607 nm, respectively. The slight blue-shifted emission of tFSeBN relative to tFSBN can be attributed to the differences in intramolecular charge transfer (ICT) strength and conformational effects induced by the larger Se atom. Benefiting from their rigid locked molecular backbones, tFSBN and tFSeBN exhibit modest Stokes shifts of 42 nm/0.14 eV and 38 nm/0.14 eV, along with relatively narrow FWHMs of 56 nm/0.18 eV and 48 nm/0.16 eV, respectively, indicating minimal conformational variation between the ground (S_0_) and S_1_ states. These heavy-atom-containing MR emitters can be further red-shifted through rational framework modification, such as replacing the tert-butylcarbazole units with derivatives featuring stronger electron-donating capability or more extended π-conjugation. Additionally, the solvatochromic behavior of these emitters was studied by varying solvent polarity from n-hexane to dichloromethane (Fig. S17). Compared to conventional TADF materials^34,35^, all three emitters exhibit relatively minor solvatochromic shifts, indicating that their emission remains predominantly governed by the SRCT effect. The Δ*E*_ST_ values estimated from the onset energies of the low-temperature fluorescence and phosphorescence spectra decrease from 0.12 eV in tFBN to 0.06 eV and 0.08 eV for tFSBN and tFSeBN, respectively. Such small energy gaps are favorable for promoting efficient RISC and exciton harvesting^36,37^.

**Table 1:** Photophysical data of the emitters

Emitter	*λ*_abs_^a^ [nm]	*λ*_em_^a^ [nm]	FWHM^a^ [nm/eV]	Δ*E*_ST_^b^ [eV]	*Ф*_PL_^c^ [%]	*τ*_PF_^d^ [ns]	*τ*_DF_^d^ [μs]	*k*_r_^e^ [10^7 ^s^−1^]	*k*_nr_^e^ [10^6 ^s^−1^]	*k*_ISC_^e^ [10^7 ^s^−1^]	*k*_RISC_^e^ [10^4 ^s^−1^]
tFBN	493	508	25/0.12	0.12	95	11.96	127.3	6.92	3.64	1.05	0.90
tFSBN	579	621	56/0.18	0.06	98	17.97	43.59	3.89	0.79	1.59	3.21
tFSeBN	569	607	48/0.16	0.08	98	5.57	4.53	5.19	1.06	12.6	74.8

^a^Peak of absorption (*λ*_abs_) and fluorescence (*λ*_em_) spectra, as well as full width at half maximum (FWHM) in toluene (1 × 10^−5^ M, 298 K)

^b^S_1_–T_1_ energy gap (Δ*E*_ST_) determined from the onset of low-temperature fluorescence and phosphorescence spectra measured in toluene (1 × 10^−5^ M, 77 K)

^c^Absolute photoluminescence quantum yield (*Φ*_PL_)

^d^Lifetimes of prompt fluorescence (*τ*_PF_) and delayed fluorescence (*τ*_DF_)

^e^Rate constants of singlet radiative decay (*k*_r_), non-radiative decay (*k*_nr_), intersystem crossing (*k*_ISC_), reverse intersystem crossing (*k*_RISC_) measured in 1 wt% DMIC-TRZ-doped films

To further investigate their photophysical behavior in the solid state, 1 wt% doped films of tFBN, tFSBN, and tFSeBN in DMIC-TRZ were prepared^38^. The corresponding emission maxima are observed at 509, 622, and 611 nm (Fig. S18). Notably, both tFSBN and tFSeBN achieve near-unity *Φ*_PL_ values (98%) higher than tFBN (95%), reflecting exceptional suppression of non-radiative decay (Fig. 3e). Transient PL measurements reveal bi-exponential decays, with nanosecond-scale prompt fluorescence and microsecond-scale delayed fluorescence (Fig. S19). Temperature-dependent transient PL decay curves further confirm the TADF nature of the tFSBN and tFSeBN (Fig. S20). Upon introducing heavier atoms, the delayed lifetime (*τ*_DF_) is significantly shortened, and the delayed component becomes more dominant: tFBN (127.3 μs/13%), tFSBN (43.6 μs/29%) and tFSeBN (4.5 μs/70%). The key exciton dynamics constants were determined by a classical methodology^39^ and compared in Fig. 3f. A pronounced enhancement in both intersystem crossing (ISC) and RISC rates was observed in the heavy-atom containing derivatives. In particular, the *k*_RISC_ values increase from tFBN (9.0 × 10^3^ s^−1^) to tFSBN (3.2 × 10^4^ s^−1^), and further to tFSeBN (7.5 × 10^5^ s^−1^), representing 3.6-fold and 83.3-fold enhancements, respectively. This remarkable increase in *k*_RISC_ of tFSeBN is attributed to the synergistic effect of Δ*E*_ST_ reduction and SOC enhancement. Such accelerated triplet-to-singlet conversion in tFSeBN is expected to mitigate efficiency roll-off under high brightness, underscoring its potential in high-performance red MR-TADF devices.

**Fig. 4:**
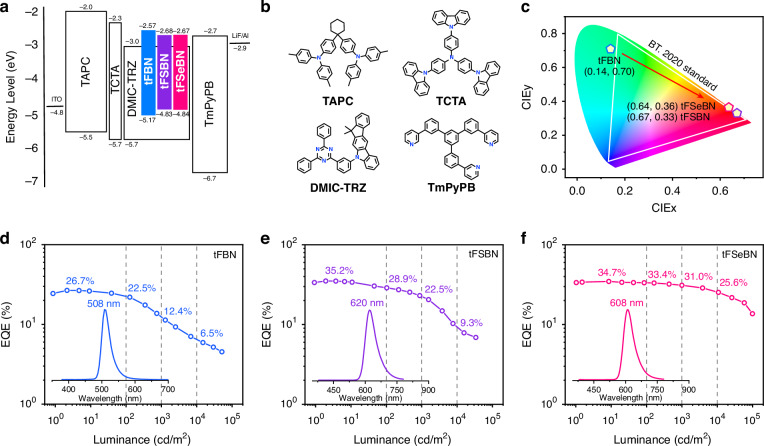
Electroluminescence performances. **a** Device configuration and the energy level diagrams. **b** The chemical structures used for the respective layers. **c** Electroluminescence color coordinates on the CIE 1931 color space. EQE versus luminance characteristics of the non-sensitized OLEDs based on **d** tFBN, **e** tFSBN, and **f** tFSeBN at the dopant concentration of 1 wt% (insert: EL spectra of the devices at the luminance of 100 cd m^−2^)

To evaluate the electroluminescence (EL) performance of the emitters, the non-sensitized OLEDs were fabricated following the optimized architecture: [indium tin oxide (ITO)/TAPC (50 nm)/TCTA (10 nm)/*x* wt% tFBN or tFSBN or tFSeBN: DMIC-TRZ (30 nm)/TmPyPB (35 nm)/LiF (1 nm)/Al (100 nm) (*x* = 1, 3, 5, 10)]. In this device configuration, ITO and Al served as the anode and cathode, respectively; TAPC (1,1-bis[(di-4-tolylamino)phenyl]cyclohexane), TCTA (tris(4-carbazolyl-9-ylphenyl)amine), and TmPyPB (3,3’-[5’-[3-(3-pridinyl)phenyl][1,1’:3’,1”-terphenyl]-3,3”-diyl]bispyridine) were employed as the hole transport layer (HTL), electron blocking layer (EBL), and the electron transport layer (ETL), respectively. DMIC-TRZ was applied as the host material for the emitting layer (EML) owing to its balanced hole and electron transport properties, enabling effective charge recombination and reducing exciton quenching^38^. The energy levels of the corresponding materials and the key EL parameters are provided in Fig. 4 and Table 2.

**Table 2:** Summary of EL data of devices (DMIC-TRZ as host in EML)

Emitter	*λ*_EL_^a^ [nm]	FWHM^b^ [nm/eV]	*V*_on_^c^ [V]	*L*_max_^d^ [cd m^−2^]	CE_max_^e^ [cd A^−1^]	PE_max_^f^ [lm W^−1^]	EQE_max/100/1000/10,000_^g^ [%]	CIE^h^ (*x*, *y*)
1 wt% tFBN	508	29/0.14	2.7	51,440	83.7	93.9	26.7/22.5/12.4/6.5	(0.14, 0.70)
1 wt% tFSBN	620	59/0.19	2.6	33,820	41.9	48.7	35.2/28.9/22.5/9.3	(0.67, 0.33)
1 wt% tFSeBN	608	57/0.19	3.1	98,710	55.9	53.2	34.7/33.4/31.0/25.6	(0.64, 0.36)

^a^EL peak wavelength

^b^FWHM of EL spectra

^c^Turn-on voltage at the luminance of 1 cd m^−2^

^d^Maximum luminance

^e^Maximum current efficiency

^f^Maximum power efficiency

^g^Maximum external quantum efficiency, and values at 100, 1000 and 10,000 cd m^−2^, respectively

^h^Commission Internationale de l’Éclairage coordinates (value taken at 100 cd m^−2^)

**Fig. 5:**
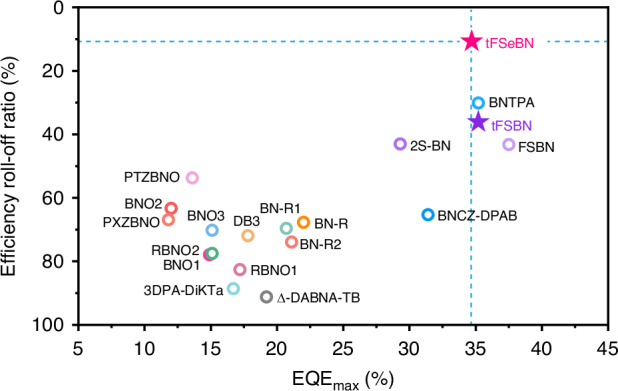
Efficiency roll-off ratio at 1000 cd m^−2^ relative to EQE_max_ versus EQE_max_ of reported red non-sensitized MR-TADF OLEDs

At the dopant concentration of 1 wt%, the OLEDs incorporating tFBN, tFSBN, and tFSeBN exhibit emission peaks at 508, 620, and 608 nm, FWHMs of 29 nm, 59 nm, and 57 nm, and corresponding CIE coordinates of (0.14, 0.70), (0.67, 0.33), and (0.64, 0.36), respectively. The FWHMs are broadened relative to their solution-state values due to the increased polarity of the host environment and host–guest interactions. The tFSBN and tFSeBN-based devices display significantly improved EQE_max_s (35.2% and 34.7%, respectively) compared to the tFBN-based device (26.7%). A more pronounced difference arises under high brightness. The tFBN and tFSBN-based devices show low EQEs of 12.4% and 22.5% at 1000 cd m^−2^, and 6.5% and 9.3% at 10,000 cd m^−2^, respectively. In contrast, tFSeBN-based device maintains high EQEs of 31.0% at 1000 cd m^−2^ and 25.6% at 10,000 cd m^−2^, corresponding to 10.7% and 26.2% roll-off ratios, respectively, relative to its EQE_max_. This ultra-low efficiency roll-off could be attributed to the very short *τ*_DF_ (4.5 μs) and fast RISC process (*k*_RISC_ = 7.5 × 10^5^ s^−1^) for tFSeBN, which facilitates the efficient triplet exciton utilization and inhibits bimolecular exciton deactivations through triplet–triplet annihilation (TTA) and singlet–triplet annihilation (STA) processes. We highlight that the tFSeBN-based device delivers a state-of-the-art EQE_max_ and the lowest efficiency roll-off at 1000 cd m^−2^ among the reported red non-sensitized MR-TADF devices (Fig. 5 and Table S6). Moreover, this device’s performance is comparable with or even surpasses that of noble metal-sensitized red MR devices (Table S6). Moreover, all devices demonstrate excellent spectral stability upon enhanced operating voltages (Fig. S21). Furthermore, these three emitters show strong resistance to ACQ effects. As the doping concentration increases from 1 to 10 wt%, all three emitters display minimal spectral shifts and FWHM variations, along with robust luminescence efficiency, benefiting from the large steric hindrance provided by the bulky tert-butyl-substituted fluorene units (Figs. S22–S24 and Tables S7–S9).

**Fig. 6:**
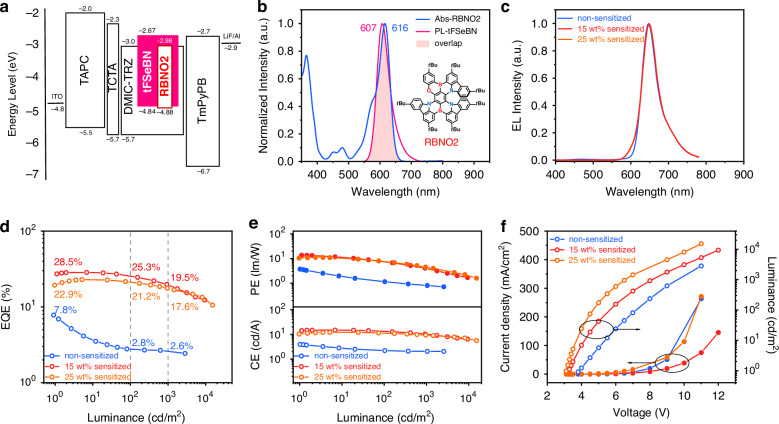
Electroluminescence performances of HF OLEDs. **a** Device configuration and the energy level diagrams. **b** UV–vis absorption of RBNO2 and fluorescence spectrum of tFSeBN in toluene solution (1 × 10^−5^ M, 298 K). Filled area reveals the overlap of them (insert: The chemical structure of RBNO2). **c** EL spectra of the devices at the luminance of 100 cd m^−2^. **d** EQE versus luminance characteristics. **e** Current efficiency (CE) versus luminance and power efficiency (PE) versus luminance curves. **f** Current density and luminance versus driving voltage characteristics

Taking advantage of its fast *k*_RISC_, near-unity *Φ*_PL_, and anti-aggregation characteristics, tFSeBN was further employed as a red MR-TADF sensitizer in HF OLEDs. As a proof of concept, RBNO2 was selected as the terminal emitter, featuring a red emission at 645 nm with a FWHM of 39 nm (0.11 eV) in toluene. The HF devices retained the same architecture as the former devices, except for the EML, which adopted a 1 wt% RBNO2:*x* wt% tFSeBN: DMIC-TRZ doping system (*x* = 15, 25) (Fig. 6a). A non-sensitized control device with only 1 wt% RBNO2 doped in DMIC-TRZ as the EML was also fabricated for comparison. As depicted in Fig. 6b, the emission spectrum of tFSeBN exhibits strong overlap with the absorption band of RBNO2, thus promoting efficient FET process in the HF devices^40^. Importantly, the bulky tert-butyl-substituted spiro-fluorene unit in tFSeBN introduces strong steric hindrance, increasing intermolecular distances and limiting the short-range orbital overlap required for Dexter energy-transfer (DET). Combined with the low RBNO₂ doping level (1 wt%), the undesired DET pathway is effectively suppressed. The HF devices display a dominant red emission at 648 nm (FWHM = 48 nm/0.14 eV) originated from RBNO2 with CIE coordinates of (0.70, 0.30), approaching the BT. 2020 standard (Fig. 6c and Table 3). To assess the effectiveness of sensitization, we fitted the EL spectrum of the HF devices using the EL spectra of the tFSeBN-only device and RBNO2-only device (Fig. S25). The result indicates that the HF devices present a high proportion of EL emission that comes from the terminal dopant RBNO2 (97%), confirming an efficient FET energy-transfer process. Due to the weak TADF character of RBNO2 with extremely slow RISC process, the non-sensitized device exhibited an EQE_max_ of only 7.8% and the EQE dropped to 2.8% at the brightness of 100 cd m^−2^ (Fig. 6d). In contrast, the HF devices doped with 15 wt% and 25 wt% tFSeBN achieve remarkable EQE_max_s of 28.5% and 22.9%, while retaining high EQEs of 19.5% and 17.6% at 1000 cd m^−2^, respectively. The HF devices could simultaneously take advantage of the fast-radiative decay rate (*k*_r_) and high color purity of RBNO2, as well as the excellent photophysical properties, including high *Φ*_PL_, rapid *k*_r_, and *k*_RISC_ by the tFSeBN. This work demonstrates the first application of a red MR-TADF molecule as a sensitizer in HF OLEDs. Owing to its well-positioned emission peak and favorable spectral overlap with the absorption spectra of many red MR-TADF emitters, tFSeBN holds great potential for broader adoption in future HF display technologies.

**Table 3:** Summary of EL data of HF devices (DMIC-TRZ: *x* wt% tFSeBN (*x* = 0, 15, 25): 1 wt% RBNO2 as EML)

*x* wt% (tFSeBN)	*λ*_EL_^a^ [nm]	FWHM^b^ [nm/eV]	*V*_on_^c^ [V]	*L*_max_^d^ [cd m^−2^]	CE_max_^e^ [cd A^−1^]	PE_max_^f^ [lm W^−1^]	EQE_max/100/1000_^g^ [%]	CIE^h^ (*x*, *y*)
0	648	47/0.14	3.8	2826	3.4	2.2	7.8/2.8/2.6	(0.69, 0.29)
15	648	48/0.14	3.3	9393	14.9	13.6	28.5/25.3/19.5	(0.70, 0.30)
25	648	48/0.14	3.1	15,240	12.1	11.1	22.9/21.2/17.6	(0.70, 0.30)

^a^EL peak wavelength

^b^FWHM of EL spectra

^c^Turn-on voltage at the luminance of 1 cd m^−2^

^d^Maximum luminance

^e^Maximum current efficiency

^f^Maximum power efficiency

^g^Maximum external quantum efficiency, and values at 100 and 1000 cd m^−2^, respectively

^h^Commission Internationale de l’Éclairage coordinates (value taken at 100 cd m^−2^)

## Conclusion

In summary, this work provides an effective pathway to overcome the severe efficiency roll-off challenge in red MR-OLEDs. The Se-embedded MR framework simultaneously narrows the Δ*E*_ST_ and enhances SOC through single-atom engineering, thereby achieving intrinsically accelerated RISC and suppressed efficiency roll-off in red MR-TADF systems. The compound tFSeBN exhibits a red emission at 607 nm and delivers a record-high *k*_RISC_ of 7.5 × 10^5^ s^–^^1^ among red MR-TADF materials. OLED based on tFSeBN demonstrates outstanding EL performance, achieving a high EQE_max_ of 34.7% and maintaining 25.6% EQE even at a high brightness of 10,000 cd m^−2^, indicating ultra-low efficiency roll-off. Moreover, tFSeBN functions effectively as a sensitizer in HF devices, delivering pure-red emission with significantly improved EQE_max_, suppressed efficiency roll-off, and CIE coordinates approaching the BT.2020 standard. This contribution will greatly accelerate the development of the noble-metal-free OLED industry.

## Materials and methods

### Materials and characterization

All commercially available reagents were used directly without further purification. The materials for device fabrication were either purchased or synthesized by coauthors. UV–vis absorption spectra were measured using a UV-2600 (Shimadzu) instrument. PL spectra were recorded on a Hitachi F-4600 fluorescence spectrophotometer. The PLQYs were obtained with an absolute photoluminescence quantum yield measurement system, Hamamatsu C9920-03G, in an integrating sphere. The solution sample was bubbled with nitrogen for 10 minutes before measurement. The transient spectra were collected on an Edinburgh Fluorescence Spectroscopy FLS1000.

### X-ray crystallography

X-ray diffraction data were collected on a Bruker D8 Quest diffractometer. A suitable crystal was selected and mounted on a CCD area detector diffractometer. The crystal was kept at 273.15 K during data collection. All crystallographic information in CIF format has been deposited at the Cambridge Crystallographic Data Center (CCDC) under deposition numbers 2469420 for tFBN, 2469448 for tFSBN, and 2469421 for FSeBN via www.ccdc.cam.ac.uk/data_request/cif.

### Theoretical calculations

The calculations were performed using the Gaussian 16 package^41^, employing the density functional theory (DFT) and time-dependent density functional theory (TD-DFT) methods. The B3LYP functionals were utilized. The spin-component scaling second-order approximate coupled-cluster (SCS-CC2) method with cc-pVDZ basis set was also employed to calculate the energy levels of singlets and triplets^42^. NTO analysis was performed using the Multiwfn package^43,44^. The drawing of HOMO, LUMO, and the hole and electron distribution plot were completed with VMD 1.9.4^45^. The spin-orbit coupling matrix elements (SOCEMs) were calculated in B3LYP/6-31 G (d, p) level using the Orca 4.2.1 package^46^. The Cartesian Coordinates of three MR molecules are presented in Table S10.

### Device fabrication and characterization

OLEDs were fabricated on the ITO-coated glass substrates with multiple organic layers sandwiched between the transparent bottom indium-tin-oxide (ITO) anode and the top metal cathode. Before device fabrication, the ITO glass substrates were pre-cleaned carefully. All material layers were deposited by vacuum evaporation in a vacuum chamber with a base pressure of 10^−6^ torr. The deposition rate of organic layers was kept at 0.1–0.2 nm s^−1^. The doping was conducted by co-evaporation from separate evaporation sources with different evaporation rates. The EL spectrum, CIE coordinates and luminance intensity of OLEDs were recorded by Photo Research PR655; meanwhile, the current density (*J*) and driving voltage (*V*) were recorded by Keithley 2400. By assuming Lambertian distribution, EQE was estimated according to brightness, electroluminescence spectrum and current density.

## Supplementary information

Supporting Information
