# Supplementary material for: Red OLED with efficiency of 25.6% at 10,000 cd m−2 based on selenium embedding multiple resonance framework
Source: Light Sci Appl. 2026 Apr 8;15:191. doi: 10.1038/s41377-026-02220-w (PMC13062009; doi:10.1038/s41377-026-02220-w)
Supplement: Supplementary file 1 — Supporting Information [file 41377_2026_2220_MOESM1_ESM.pdf]

Supplemental information

Red OLED with Efficiency of 25.6% at 10,000 cd m^{-2} Based on Selenium Embedding Multiple Resonance Framework

Yexuan Pu[1], Xinliang Cai[1], Chenglong Li[1], Baoyan Liang[2,*], Hai Bi[2,*] and Yue Wang[1,*]

1. Y. Pu, Dr. X. Cai, Prof. C. Li, and Prof. Y. Wang
   State Key Laboratory of Supramolecular Structure and Materials, College of Chemistry, Jilin University, Changchun 130012, P. R. China.
   E-mail: yuewang@jlu.edu.cn (Y. W.)
2. Dr. B. Liang, and Dr. H. Bi
   Jihua Laboratory, 28 Huandao South Road, Foshan 528200, Guangdong Province, P. R. China
   E-mails: liangby@jihualab.ac.cn (B.L.) and bihai@jihualab.com (H.B.)

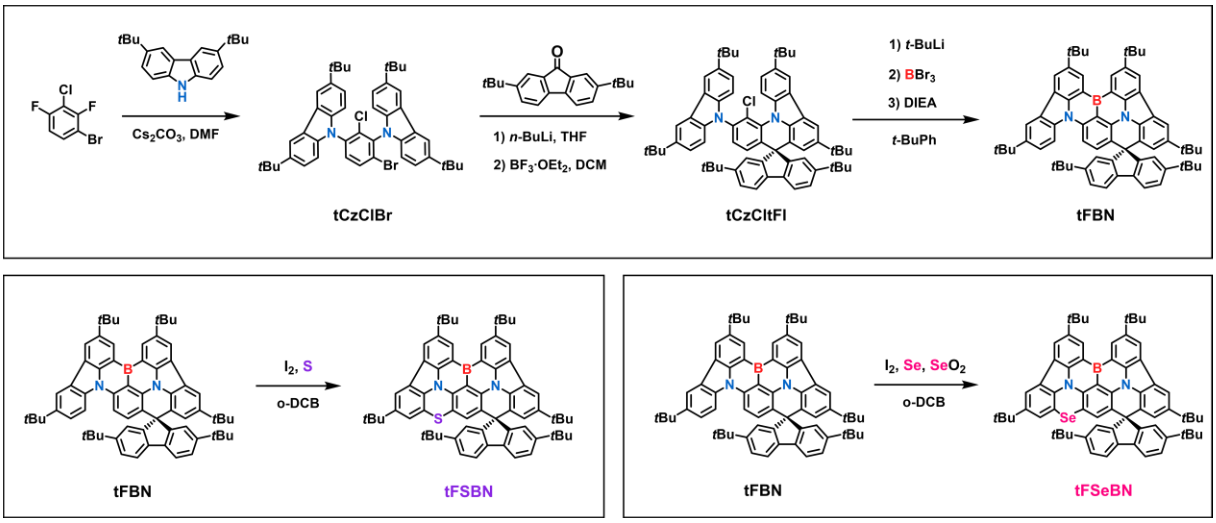

Scheme S1. Synthetic procedures of tFBN, tFSBN and tFSeBN.

Synthesis of tCzClBr: 1-bromo-3-chloro-2,4-difluorobenzene (3.41 g, 15.0 mmol), 3,6-di-*tert*-butyl-9H-carbazole (8.80 g, 31.5 mmol) and Cs_2CO_3 (14.66 g, 45.0 mmol) were added into anhydrous *N*,*N*-dimethylformamide (DMF) (150 mL) under nitrogen atmosphere. The mixture was stirred at 150 °C for 12 h. After cooling to room temperature, the mixture was poured into water (400 mL) and filtered to obtain a powder solid. The crude product was further purified by column chromatography with a mixture eluent of dichloromethane/petroleum ether (1:5), resulting in a white solid (10.30 g, 92%). ^{1}H NMR (500 MHz, Chloroform-*d*) δ 8.19 – 8.11 (m, 4H), 7.92 (d, *J* = 8.5 Hz, 1H), 7.57 (d, *J* = 8.6 Hz, 1H), 7.50 (d, *J* = 8.5 Hz, 4H), 7.10 (d, *J* = 8.5 Hz, 2H), 7.01 (d, *J* = 8.5 Hz, 2H), 1.47 (d, *J* = 5.1 Hz, 36H). MALDI-TOF MS: Calculated: 746.28, Found: 747.39.

Synthesis of tCzCltFl: The solution of *n*-butyllithium (*n*-BuLi) in *n*-hexane (5.0 mL, 2.5 M, 12.5 mmol) was added slowly to a solution of **tCzClBr** (7.46 g, 10.0 mmol) in anhydrous tetrahydrofuran (THF) (120 mL) at -78 °C under nitrogen atmosphere. After stirring at -78 °C for 2 h, a solution of 2,7-Di-tert-butyl-9H-fluoren-9-one (4.39 g, 15.0 mmol) in anhydrous THF was slowly added. Then the mixture was gradually allowed warmed to room temperature and stirred for 6 h. After quenching by 30 mL $NaHCO_3$ aqueous solution, the resulted solution was extracted with ethyl acetate and water, and the organic layer was concentrated in vacuum. The crude product was dissolved in anhydrous dichloromethane (DCM) (150 mL), and then boron trifluoride-diethyl etherate ($BF_3·OEt_2$) (3 mL) was added slowly at room temperature. The reaction mixture was stirred overnight, and slowly quenched by 30 mL $NaHCO_3$ aqueous

solution. The resulted solution was extracted with dichloromethane and water, and the organic layer was concentrated in vacuum. The product was further purified by column chromatography with a mixture eluent of dichloromethane/petroleum ether (1:5), resulting in a white solid (7.07 g, 75%). ^{1}H NMR (500 MHz, Chloroform-*d*) δ 8.13 (d, *J* = 1.9 Hz, 2H), 8.07 (d, *J* = 2.0 Hz, 1H), 7.90 (d, *J* = 1.6 Hz, 1H), 7.75 (d, *J* = 8.8 Hz, 1H), 7.71 (d, *J* = 8.0 Hz, 2H), 7.54 (dd, *J* = 8.7, 2.0 Hz, 1H), 7.47 (dd, *J* = 8.6, 1.9 Hz, 2H), 7.42 (dd, *J* = 8.0, 1.9 Hz, 2H), 7.22 (d, *J* = 1.8 Hz, 2H), 7.12 (d, *J* = 8.6 Hz, 2H), 6.97 (d, *J* = 8.3 Hz, 1H), 6.73 (d, *J* = 8.2 Hz, 1H), 6.56 (d, *J* = 1.6 Hz, 1H), 1.48 (d, *J* = 11.3 Hz, 27H), 1.20 (d, *J* = 10.4 Hz, 27H). MALDI-TOF MS: Calculated: 940.55, Found: 940.82.

Synthesis of tFBN: The solution of *tert*-butyllithium(*t*-BuLi) in pentane (10.8 mL, 1.3 M, 14.0 mmol) was added slowly to a solution of **tCzCltFl** (6.58 g, 7.0 mmol) in *tert*-butylbenzene (70 mL) at -78 °C under nitrogen atmosphere. After stirring at 60 °C for 3 h, pentane was removed in vacuum. After addition of boron tribromide (BBr_3) (3.51 g, 14.0 mmol) at 0 °C, the reaction mixture was stirred at room temperature for 1 h. *N*,*N*-Diisopropylethylamine (DIEA) (2.33 g, 18.0 mmol) was added at 0 °C and then the reaction mixture was warmed to room temperature. After stirring at 160 °C for 12 h, the reaction mixture was cooled to room temperature. 10 mL methanol was added to the reaction mixture to quench residual BBr_3.The resulted solution was extracted with dichloromethane and water, and the organic layer was concentrated in vacuum. The product was further purified by column chromatography with a mixture eluent of dichloromethane/petroleum ether (1:7), resulting in a yellow solid (3.72 g, 58%). ^{1}H NMR (600 MHz, Methylene Chloride-d_2) δ 9.16 (dd, *J* = 15.1, 1.8 Hz, 2H), 8.60 (d, *J* = 1.6 Hz, 1H), 8.52 (d, *J* = 1.9 Hz, 1H), 8.27 (d, *J* = 2.0 Hz, 1H), 8.17 (d, *J* = 8.8 Hz, 1H), 8.09 (d, *J* = 1.5 Hz, 1H), 7.93 (d, *J* = 8.7 Hz, 1H), 7.86 – 7.83 (m, 2H), 7.58 (dd, *J* = 8.8, 2.1 Hz, 1H), 7.47 (dd, *J* = 8.2, 1.8 Hz, 2H), 7.11 (d, *J* = 1.7 Hz, 2H), 6.98 (d, *J* = 8.6 Hz, 1H), 6.60 (d, *J* = 1.4 Hz, 1H), 1.70 (d, *J* = 8.4 Hz, 18H), 1.48 (s, 9H), 1.23 (s, 9H), 1.11 (s, 18H). ^{13}C NMR (151 MHz, Methylene Chloride-d_2) δ 157.0, 151.5, 147.3, 145.4, 145.0, 144.8, 142.8, 141.8, 139.7, 138.1, 137.0, 136.3, 134.0, 132.9, 129.7, 129.4, 126.8, 124.8, 124.6, 124.5, 124.5, 123.9, 123.3, 122.9, 122.7, 121.8, 121.1, 120.5, 119.5, 119.2, 117.3, 116.4, 113.8, 107.9, 35.4, 35.1, 35.0, 34.8, 34.6, 32.1, 31.9, 31.6, 31.5, 31.1. MALDI-TOF HR-MS: Calculated: 914.5710,

Found: 914.5815.

Synthesis of tFSBN: Iodine (200 mg, 0.8 mmol), sulfur (3.20 g, 100.0 mmol), and **tFBN** (1.83 g, 2.0 mmol) were dissolved in 1,2-dichlorobenzene (o-DCB) (50 mL) under nitrogen atmosphere. Then the mixture was heated to reflux and stirred for 5 days. After cooling to room temperature, the reaction mixture was directly concentrated under reduced pressure and purified by column chromatography with using a mixture eluent of dichloromethane/petroleum ether (1:4), resulting in a purple solid (1.51 g, 80%).^{1}H NMR (500 MHz, DMSO-d_6) δ 8.85 – 8.81 (m, 2H), 8.67 (s, 1H), 8.55 (s, 1H), 8.15 (s, 1H), 7.87 (d, J = 8.1 Hz, 2H), 7.78 (s, 1H), 7.46 (d, J = 7.5 Hz, 2H), 7.01 (s, 2H), 6.90 (s, 1H), 6.38 (s, 1H), 6.16 (s, 1H), 1.61 (d, J = 16.0 Hz, 18H), 1.32 (s, 9H), 1.13 (d, J = 17.8 Hz, 27H). ^{13}C NMR (151 MHz, Benzene-d_6) δ 157.2, 152.1, 148.2, 147.7, 145.2, 145.0, 141.0, 139.7, 137.5, 136.2, 135.1, 134.0, 132.8, 130.0, 125.8, 125.5, 125.4, 124.3, 124.2, 123.9, 123.7, 123.1, 122.8, 122.2, 120.7, 120.0, 118.9, 118.7, 117.3, 114.5, 112.3, 58.0, 35.5, 35.4, 35.3, 35.0, 34.9, 32.4, 32.3, 31.9, 31.7, 31.4. MALDI-TOF HR-MS: Calculated: 944.5285, Found: 944.5169. Anal. Calcd for $C_{67}H_{69}BN_2S$: C, 85.14; H, 7.36; N, 2.96; S, 3.39. Found: C, 84.87; H, 7.68; N, 2.83; S, 3.01.

Synthesis of tFSeBN: Iodine (200 mg, 0.8 mmol), selenium dioxide (222 mg, 2.0 mmol), selenium (790 mg, 10.0 mmol), and **tFBN** (1.83 g, 2.0 mmol) were dissolved in 1,2-dichlorobenzene (o-DCB) (50 mL) under nitrogen atmosphere. Then the mixture was heated to reflux and stirred for 8 hours. After cooling to room temperature, the reaction mixture was directly concentrated under reduced pressure and purified by column chromatography with using a mixture eluent of dichloromethane/petroleum ether (1:8), resulting in a pink solid (0.83 g, 42%). ^{1}H NMR (500 MHz, DMSO-d_6) δ 8.89 (d, J = 6.8 Hz, 2H), 8.72 (s, 1H), 8.62 (s, 1H), 8.18 (s, 1H), 7.89 (d, J = 9.2 Hz, 3H), 7.47 (d, J = 8.3 Hz, 2H), 7.20 (s, 1H), 7.02 (s, 2H), 6.41 (s, 1H), 6.36 (s, 1H), 1.62 (d, J = 13.0 Hz, 18H), 1.34 (s, 9H), 1.12 (d, J = 19.6 Hz, 27H). ^{13}C NMR (151 MHz, Benzene-d_6) δ 157.3, 152.1, 148.3, 147.7, 145.2, 145.0, 140.8, 140.5, 137.5, 137.3, 135.7, 134.1, 133.8, 130.9, 130.1, 130.0, 125.9, 125.4, 124.4, 124.3, 124.2, 123.7, 123.2, 122.4, 122.2, 121.4, 121.4, 120.0, 117.3, 114.9, 112.8, 106.4, 58.0, 35.5, 35.3, 34.9, 34.9, 32.4, 32.3, 31.9, 31.7, 31.3. MALDI-TOF HR-MS: Calculated: 992.4719, Found: 992.4851. Anal. Calcd for $C_{67}H_{69}BN_2Se$: C, 81.12; H, 7.01; N, 2.82. Found: C, 79.02; H, 7.27; N, 2.57.

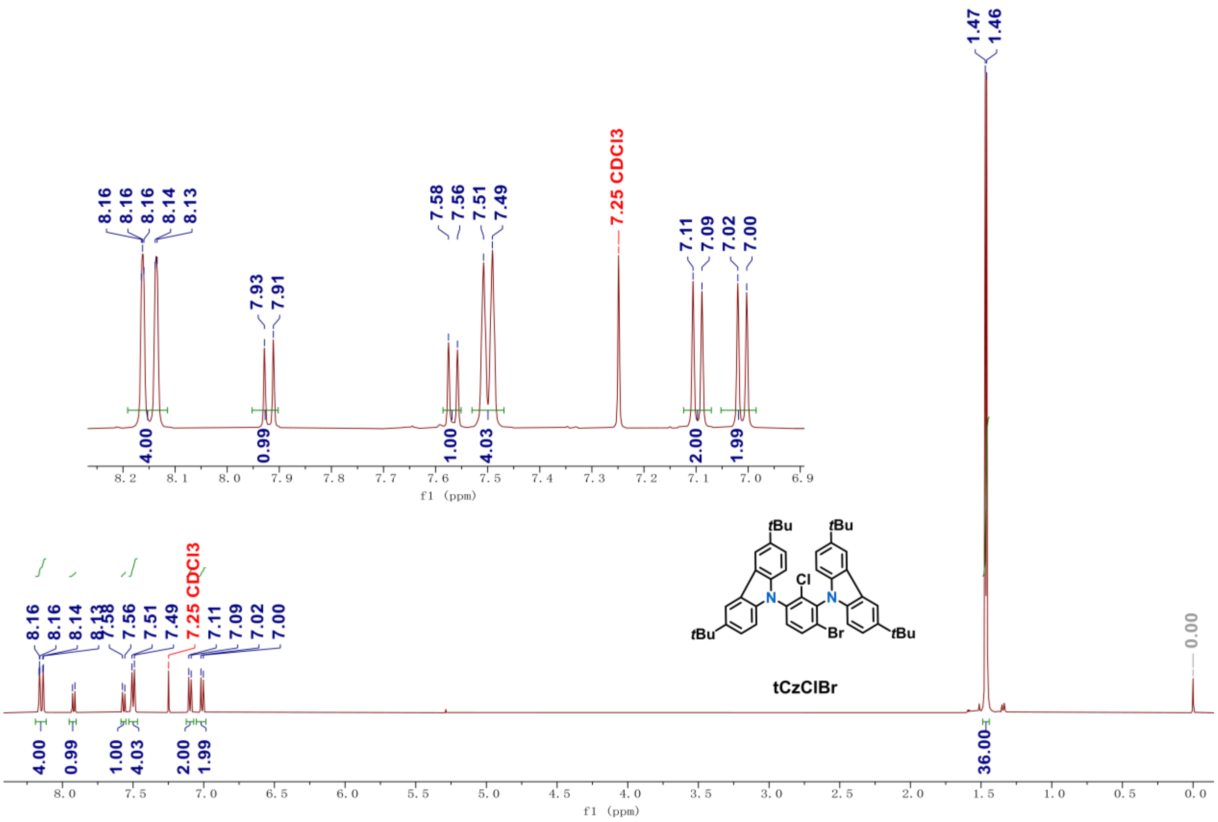

Fig. S1 ^{1}H NMR spectrum of **tCzClBr** (500 MHz, $CDCl_3$).

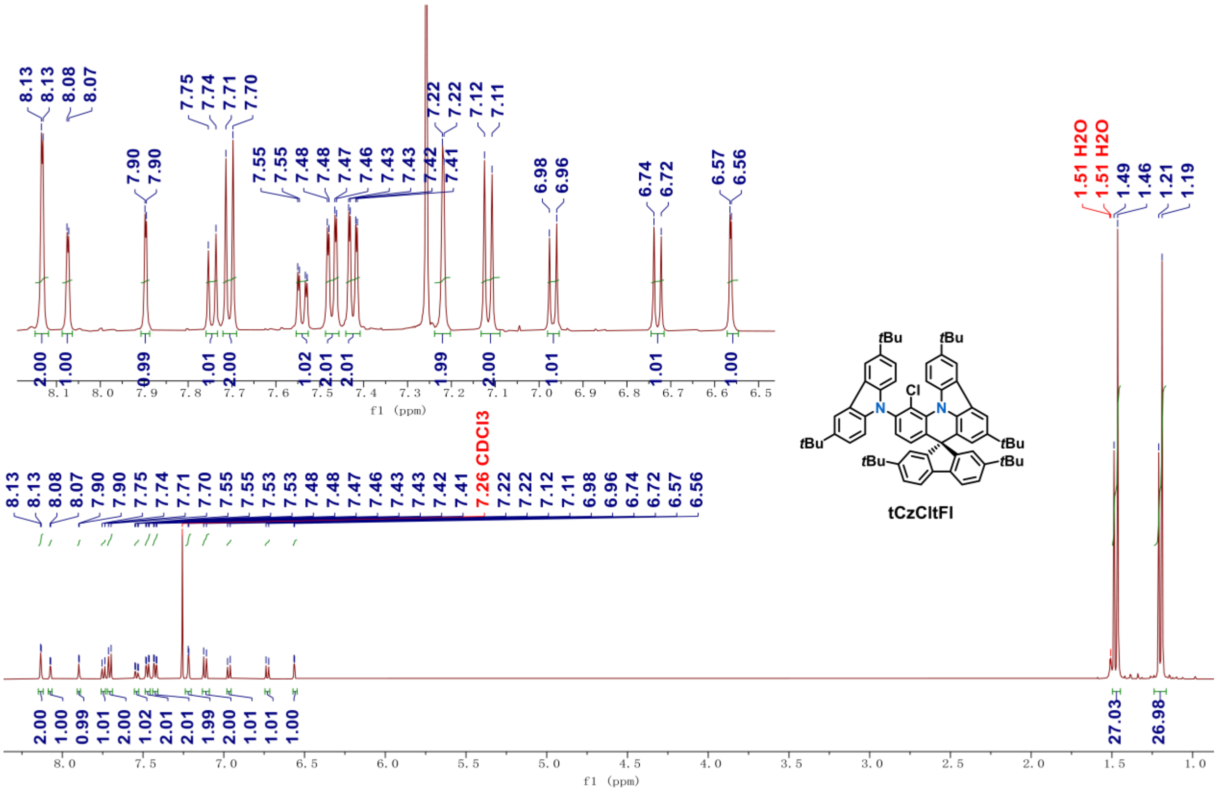

Fig. S2 ^{1}H NMR spectrum of **tCzCltFl** (500 MHz, $CDCl_3$).

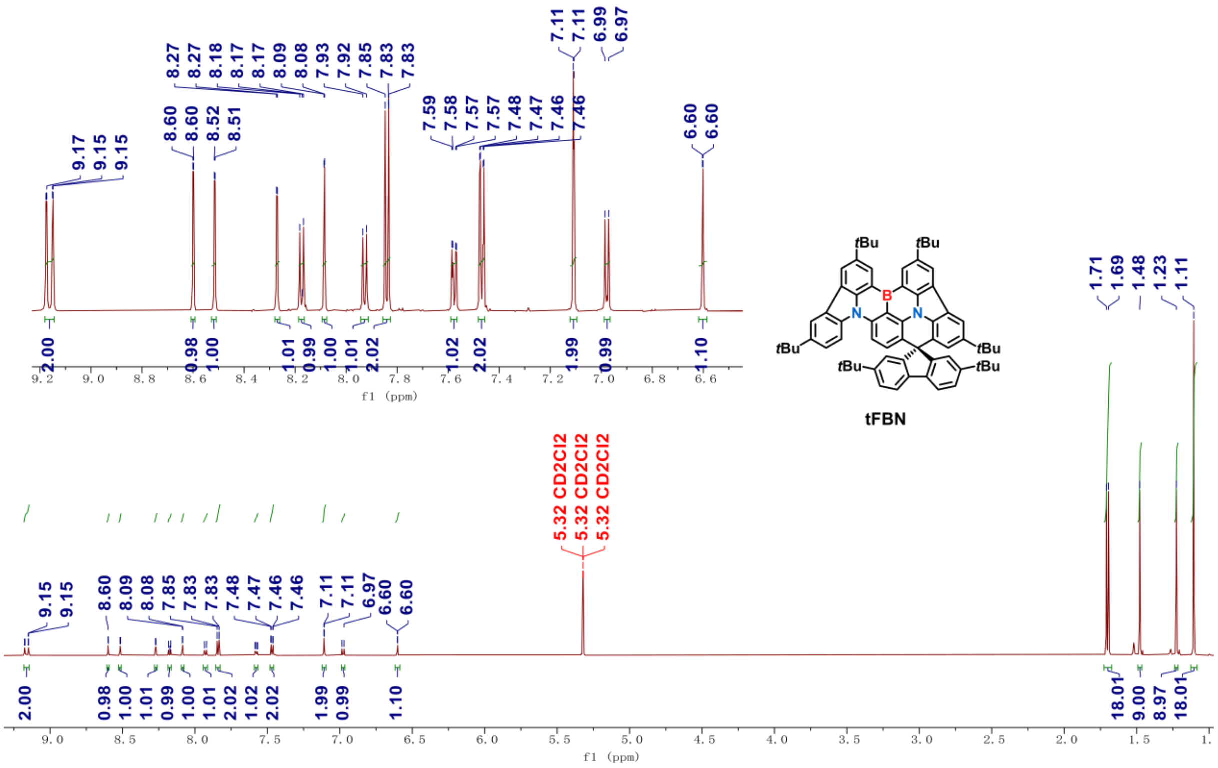

Fig. S3 ^{1}H NMR spectrum of **tFBN** (600 MHz, CD_2Cl_2).

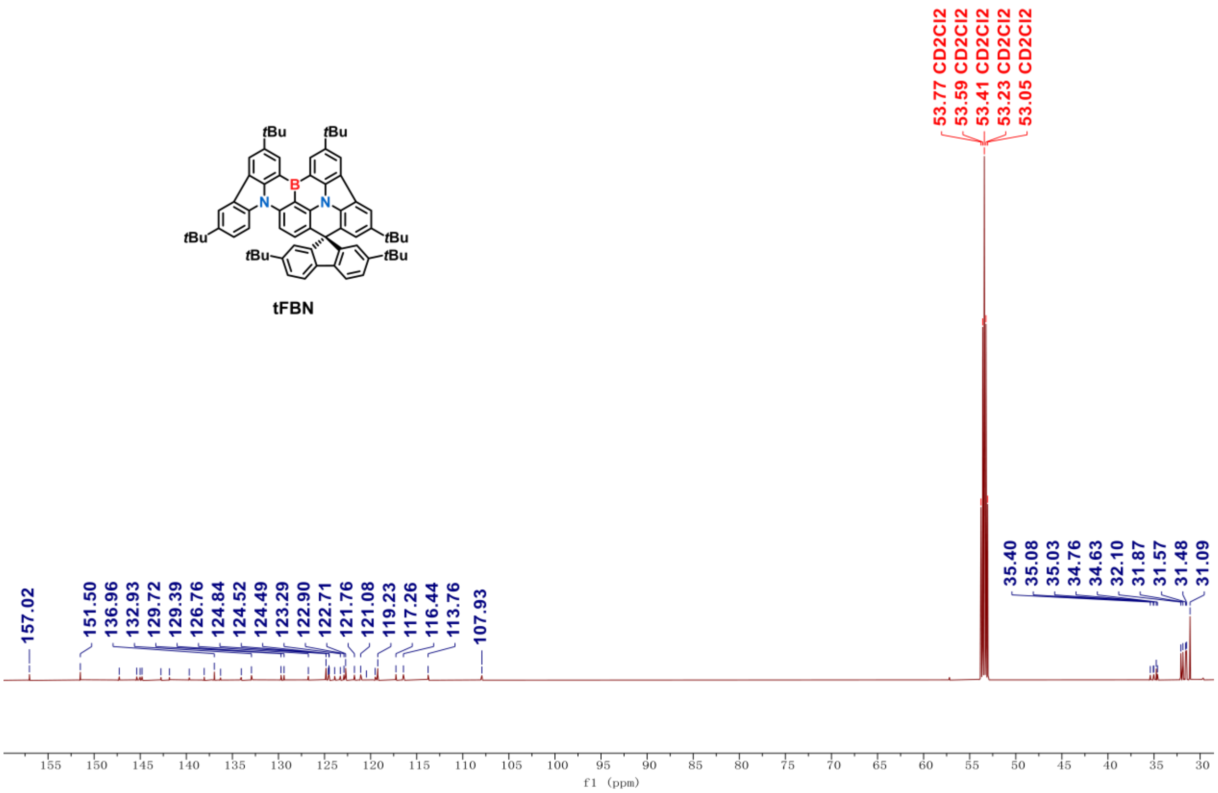

Fig. S4 ^{13}C NMR spectrum of **tFBN** (151 MHz, CD_2Cl_2).

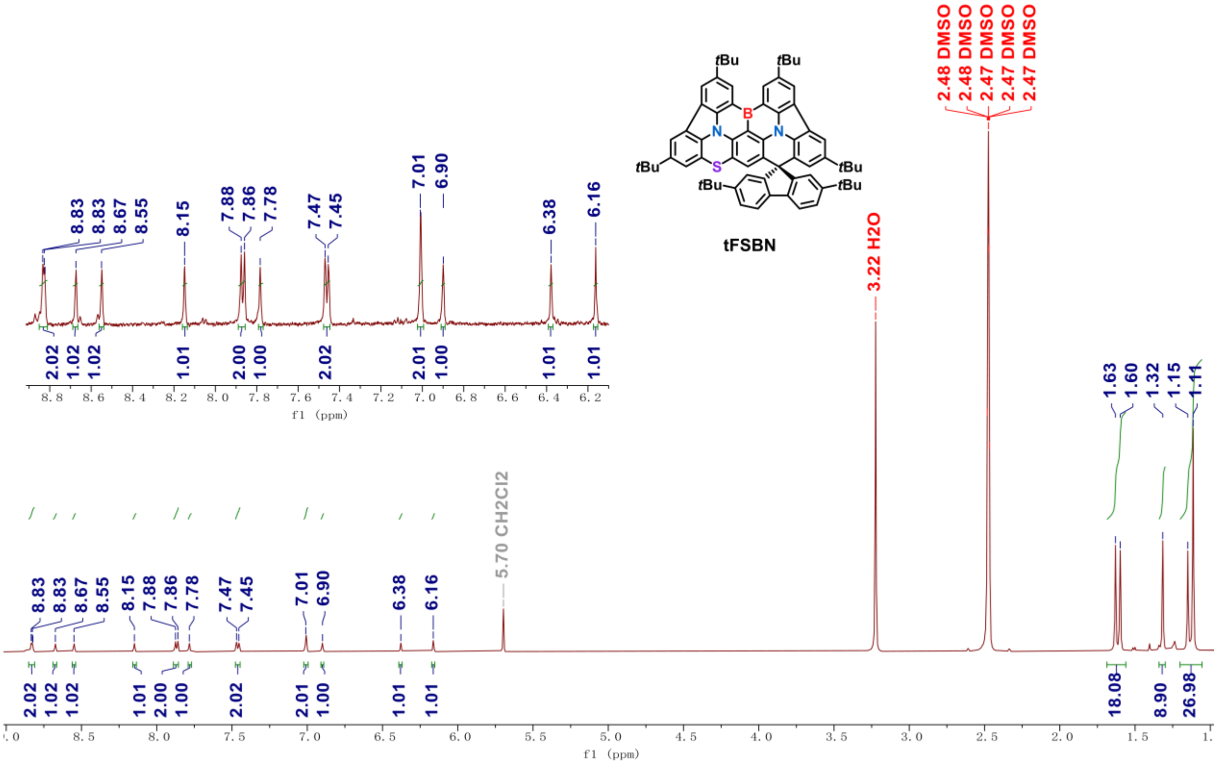

Fig. S5 ^{1}H NMR spectrum of **tFSBN** (500 MHz, DMSO-d_6 + CS_2).

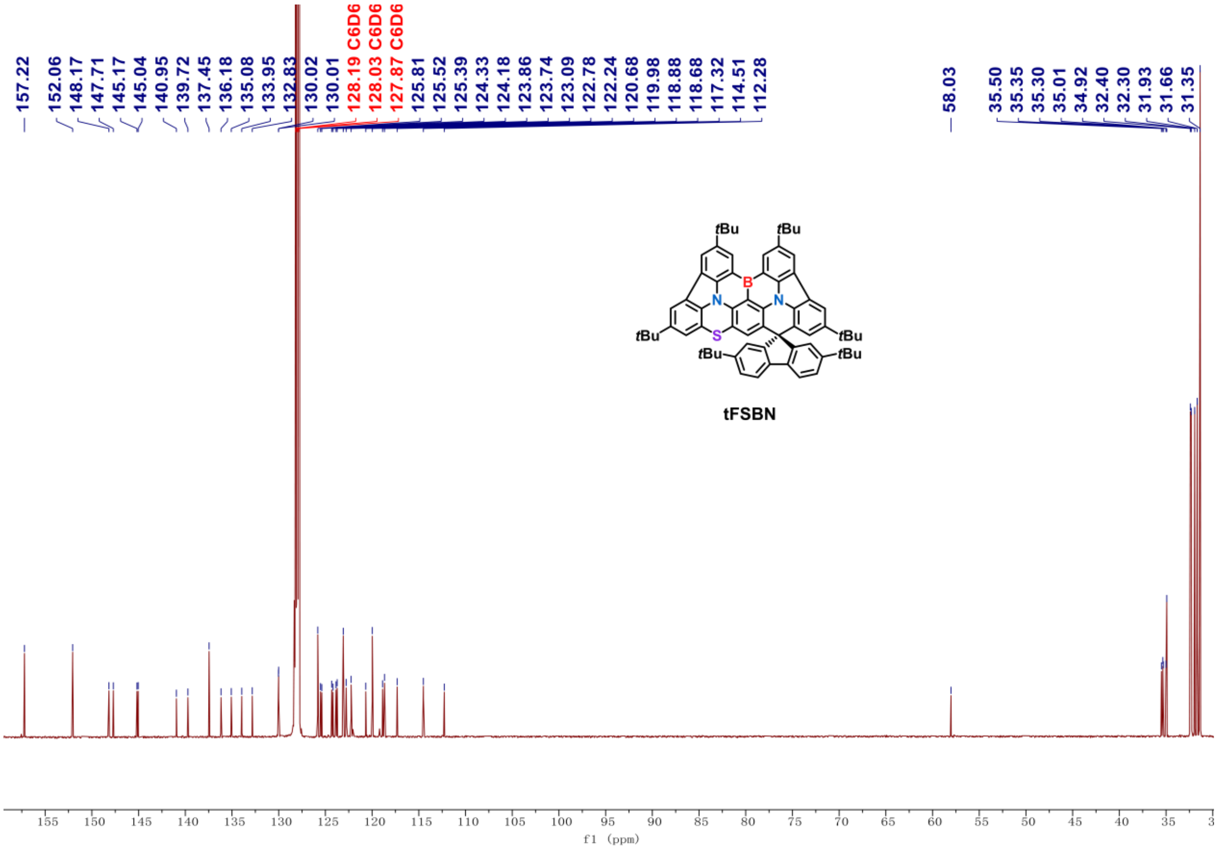

Fig. S6 ^{13}C NMR spectrum of **tFSBN** (151 MHz, C6D6).

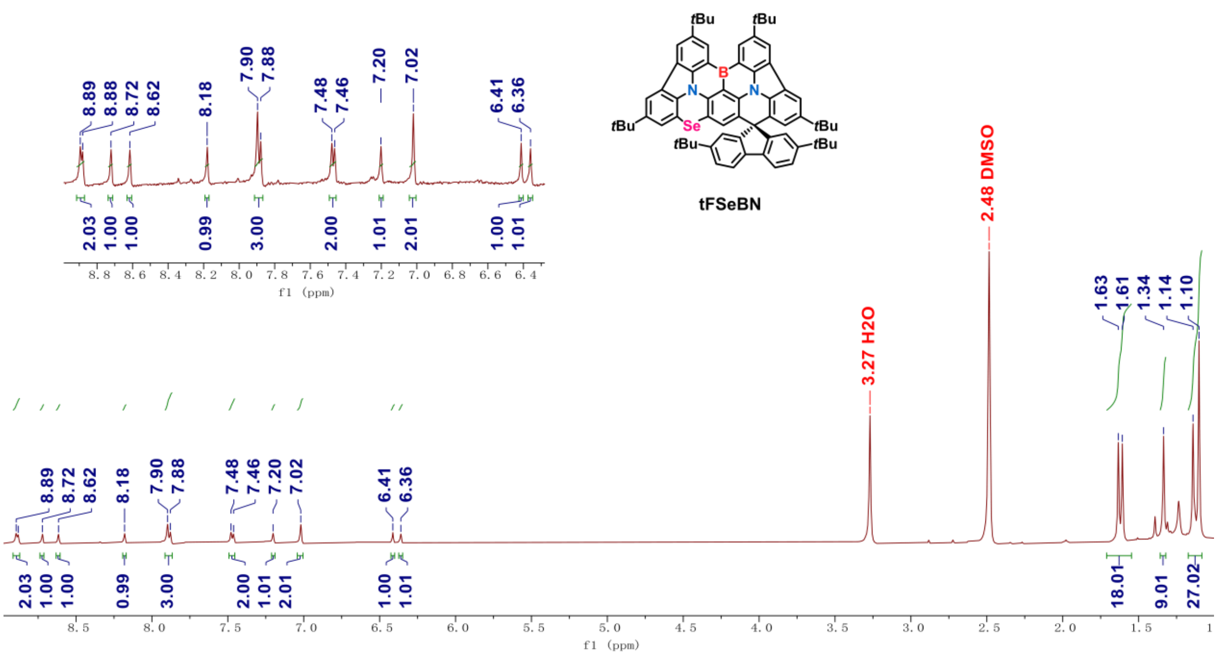

Fig. S7 ^{1}H NMR spectrum of **tFSeBN** (500 MHz, DMSO-d_6 + CS_2).

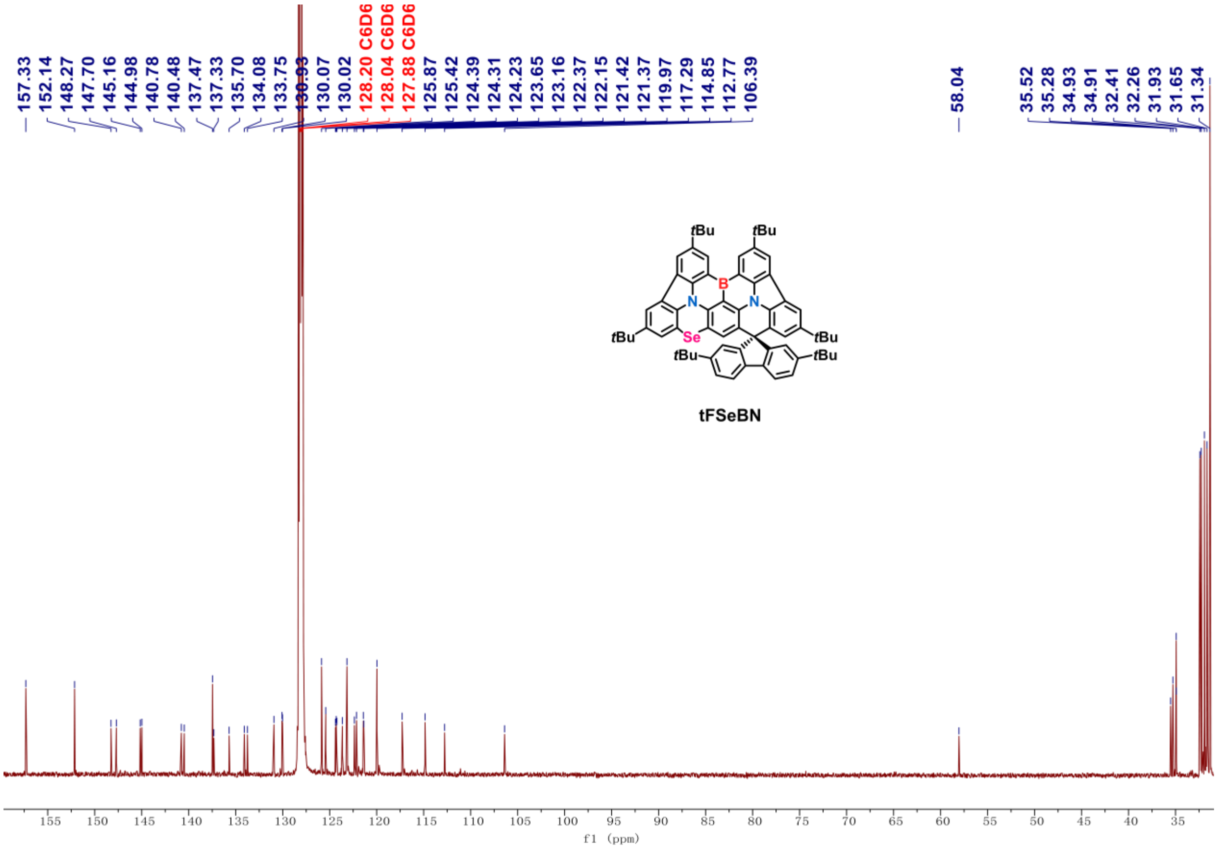

Fig. S8 ^{13}C NMR spectrum of **tFSeBN** (151 MHz, C6D6).

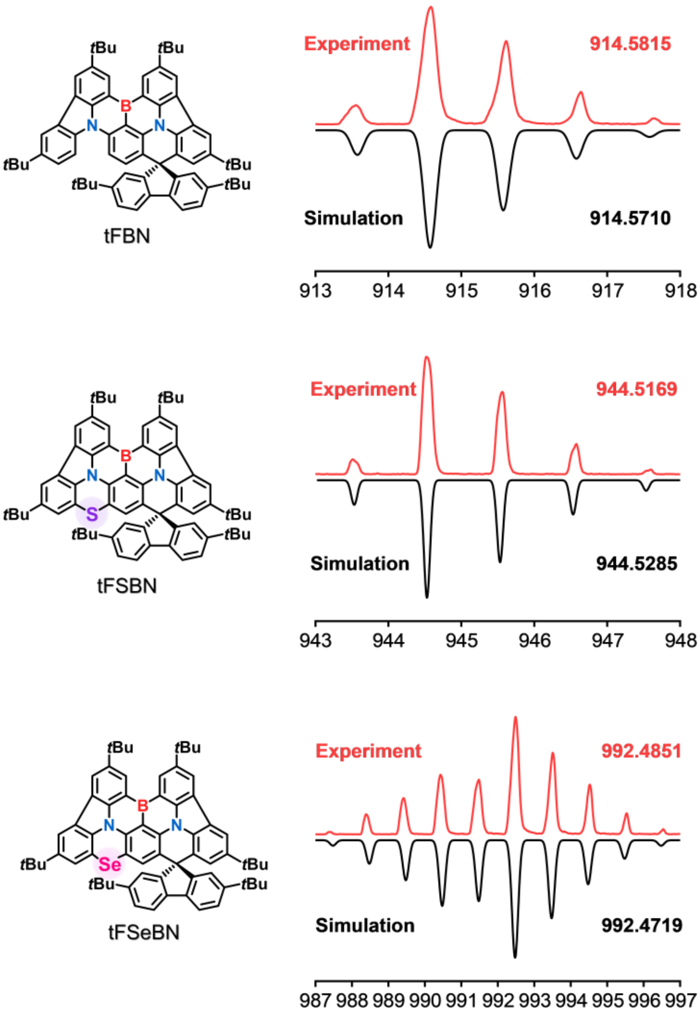

Fig. S9 HR-MS spectrum of **tFBN**, **tFSBN** and **tFSeBN**.

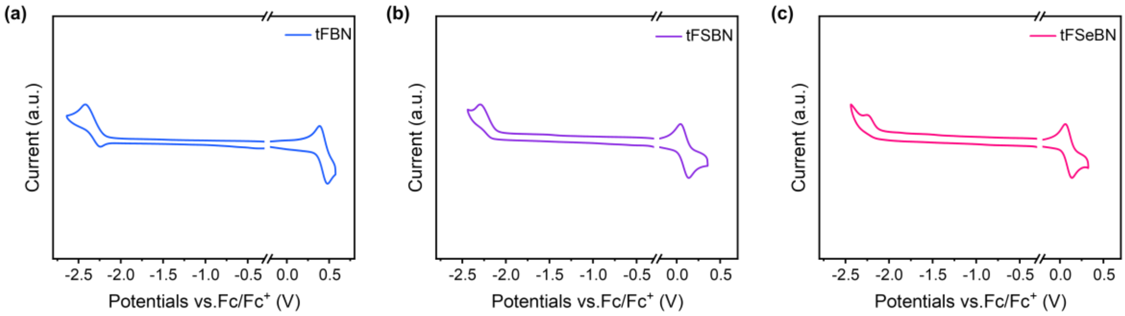

Fig. S10 Cyclic voltammograms (CV) of **tFBN**, **tFSBN** and **tFSeBN**.

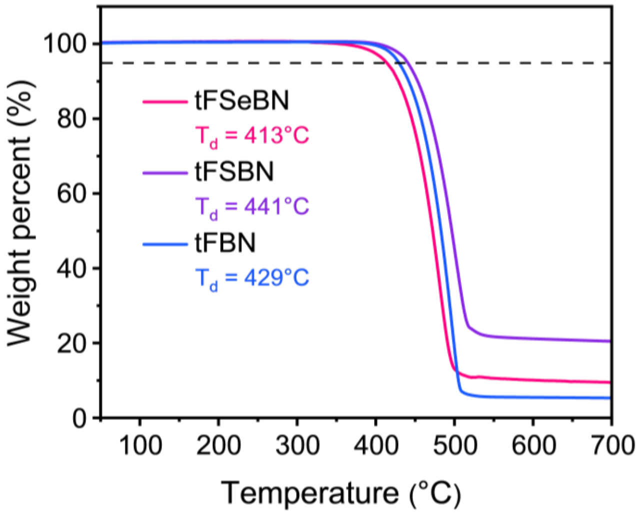

Fig. S11 Thermal gravimetric analysis (TGA) curves at a heating rate of 10 °C min^{-1}.

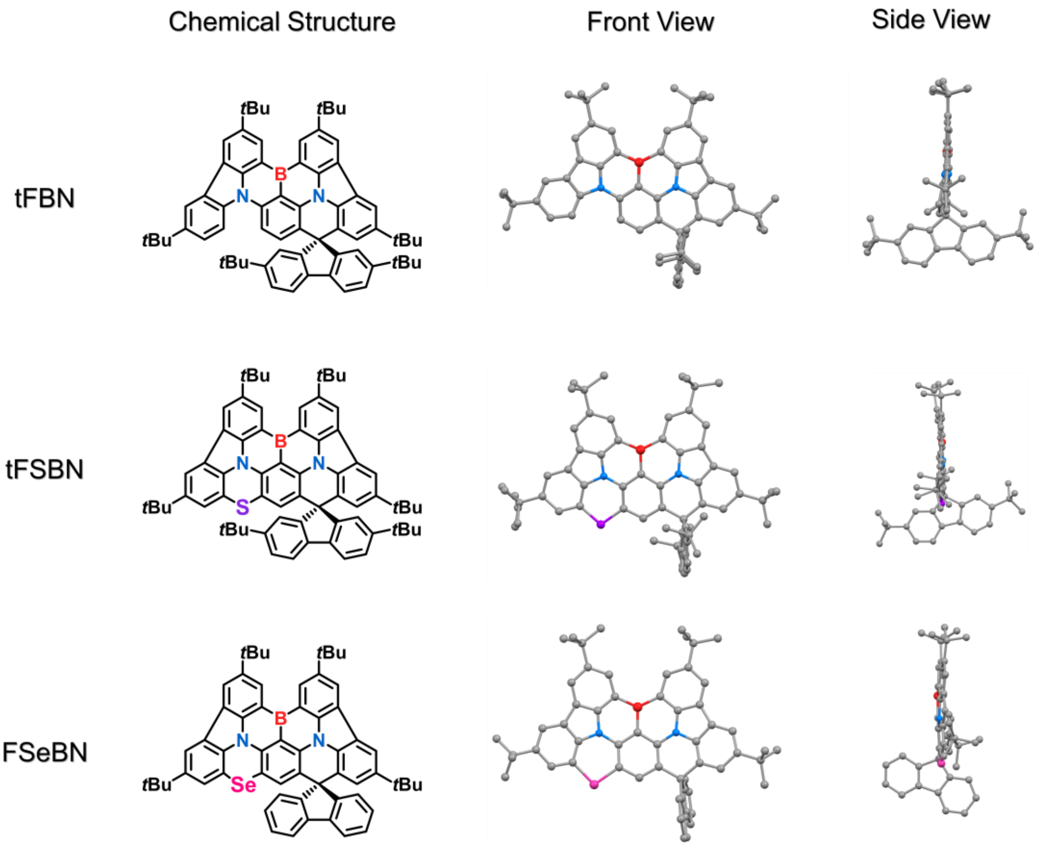

Fig. S12 Chemical structures and corresponding single crystal structures of **tFBN**, **tFSBN** and **FSeBN**. (**FSeBN** shares the same conjugated backbone as **tFSeBN**, serving as a reliable model for analyzing the geometric features of the selenium-containing MR core.)

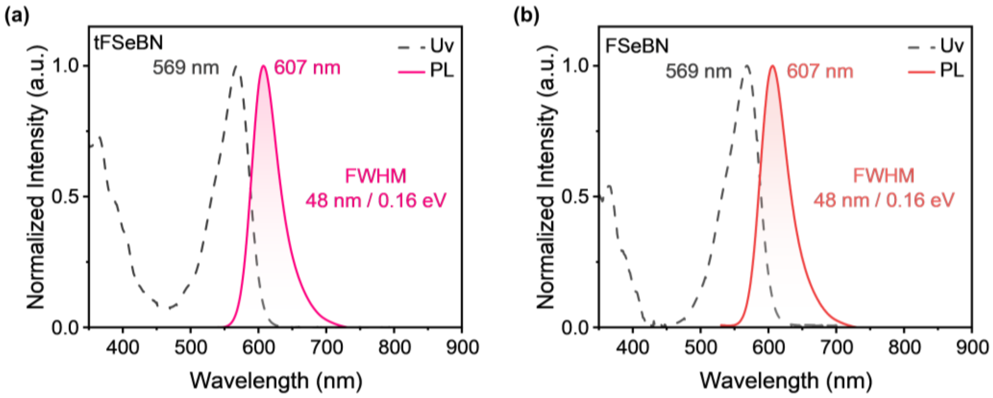

Fig. S13 The comparison of the UV–Vis absorption and PL spectra of (a) **tFSeBN** and (b) **FSeBN**.

FSeBN was employed for single-crystal analysis, whereas tFSeBN was selected for in-depth investigation and device fabrication in this work. This is because the tert-butyl-induced steric hindrance of tFSeBN not only suppresses intermolecular π-π stacking and aggregation-caused quenching but also increases intermolecular separation in hyperfluorescence devices, effectively inhibiting the unwanted Dexter energy transfer (DET) process. These combined advantages make tFSeBN well suited to serve as both the emitter and the sensitizer.

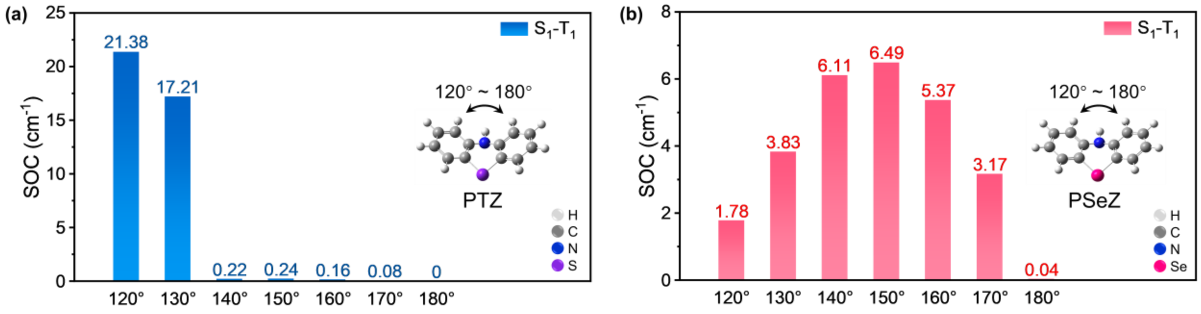

Fig. S14 SOC matrix elements based on the molecular geometries of the (a) PTZ and (b) PSeZ at different folding dihedral angles.

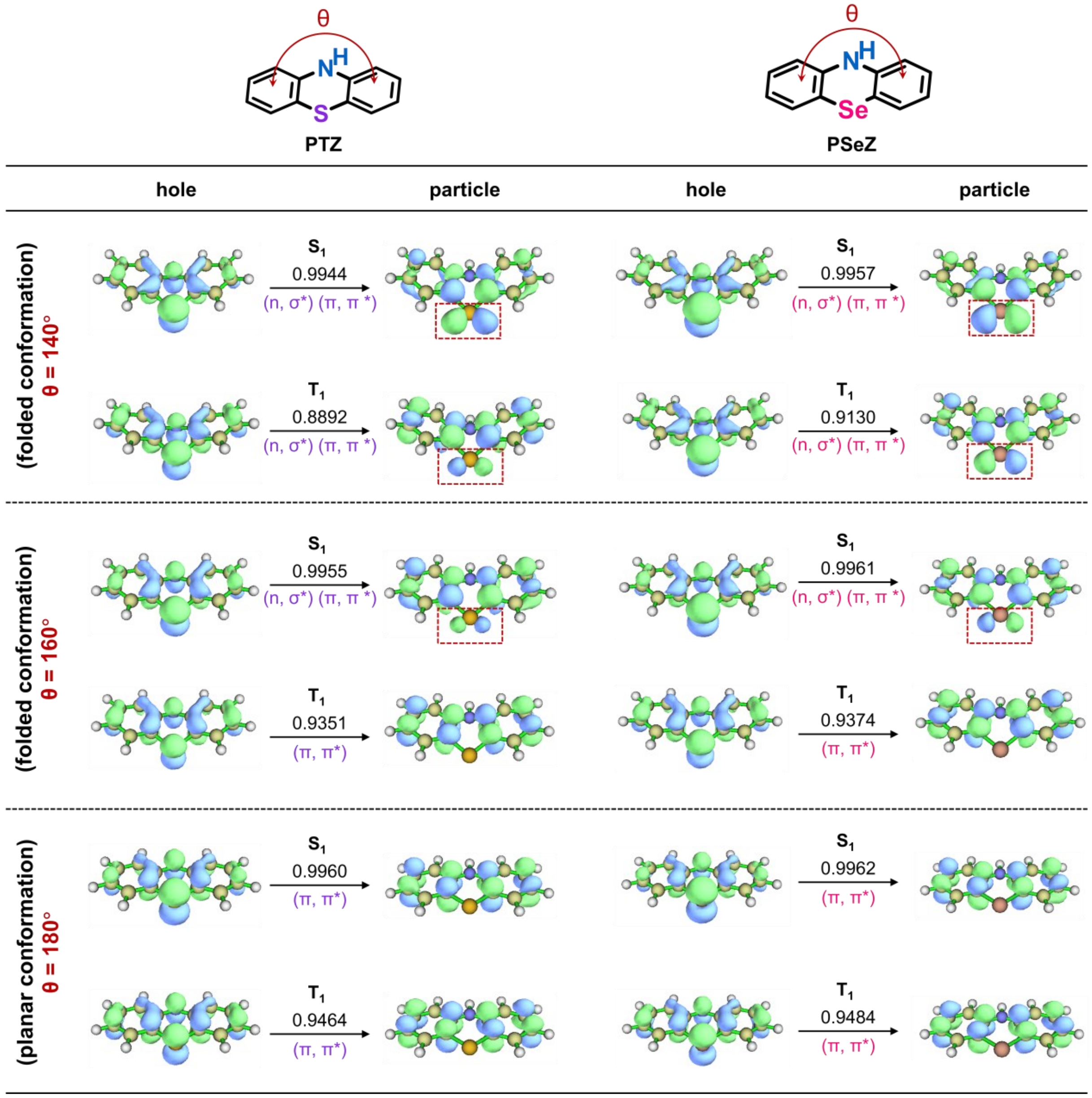

Fig. S15 NTOs of S_1 and T_1 states for PTZ and PSeZ in folded conformation (θ = 140° and 160°) and planar conformation (θ = 180°).

To investigate the crucial role of molecular conformation in modulating SOC strength in heavy atom-containing systems. We select two simplified structural models: phenothiazine (PTZ) and phenoselenazine (PSeZ), deriving from the heavy-atom-containing fragments of tFSBN and tFSeBN, respectively. The folding dihedral angles (θ) of PTZ and PSeZ were systematically varied from 180° to 120° at 10° intervals. As shown in **Fig. S14**, the SOC values in the planar conformation are nearly negligible (0.00 cm^{-1} for PTZ and 0.04 cm^{-1} for PSeZ), while in folded geometries, the SOC values achieve significant enhancement. Representative geometries (θ = 140°, 160°, 180°) were selected for natural transition orbital (NTO) analysis,

where the folded geometries allow the lone pair electron of the p_z-orbitals of the heavy atoms to be extracted from the p–π conjugation, leading to the emergence of (n, σ*) character alongside the dominant (π, π*) transitions (**Fig. S15**). This break in orbital symmetry allows for greater SOC matrix elements, consistent with El-Sayed's rule. In essence, the folded geometry provides an important structural foundation for effective activation of the heavy atom effect.

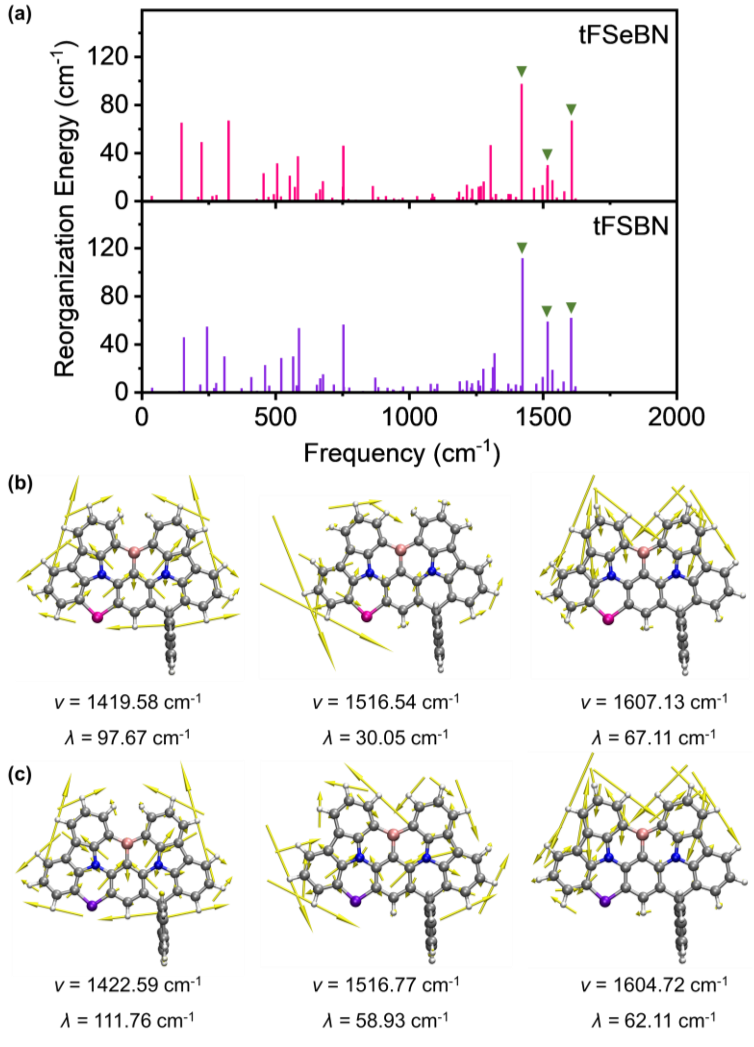

Figure S16. (a) Transition frequencies and reorganization energies of the vibrational modes contributing to the S_1-S_0 transition; The major vibrational modes for the S_1-S_0 transition of (b) **tFSeBN** and (c) **tFSBN**. (The peripheral tert-butyl moiety is removed. "v" and "λ" denote frequency and reorganization energy, respectively.)

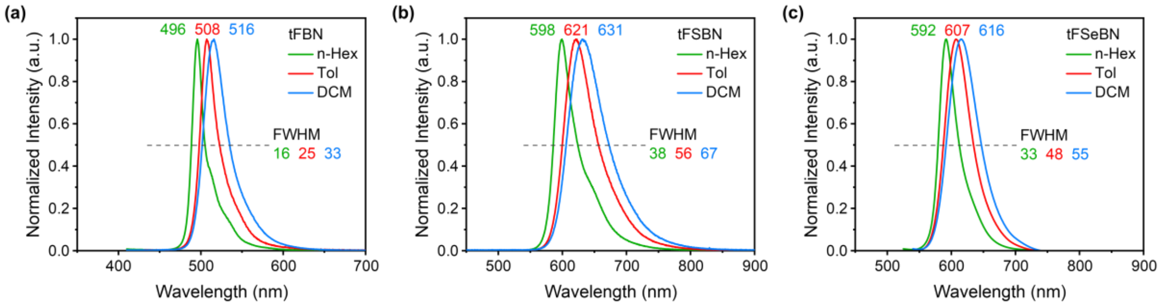

Fig. S17 Fluorescence spectra of **tFBN**, **tFSBN** and **tFSeBN** in different solvents (1×10^{-5} M, 298 K).

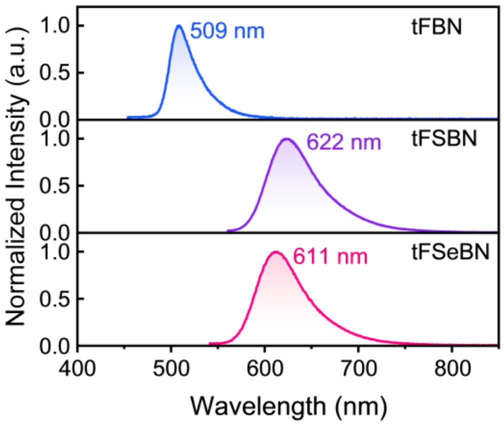

Fig. S18 Fluorescence spectra of **tFBN**, **tFSBN** and **tFSeBN** with 1 wt% doping concentration in DMIC-TRZ deposited films.

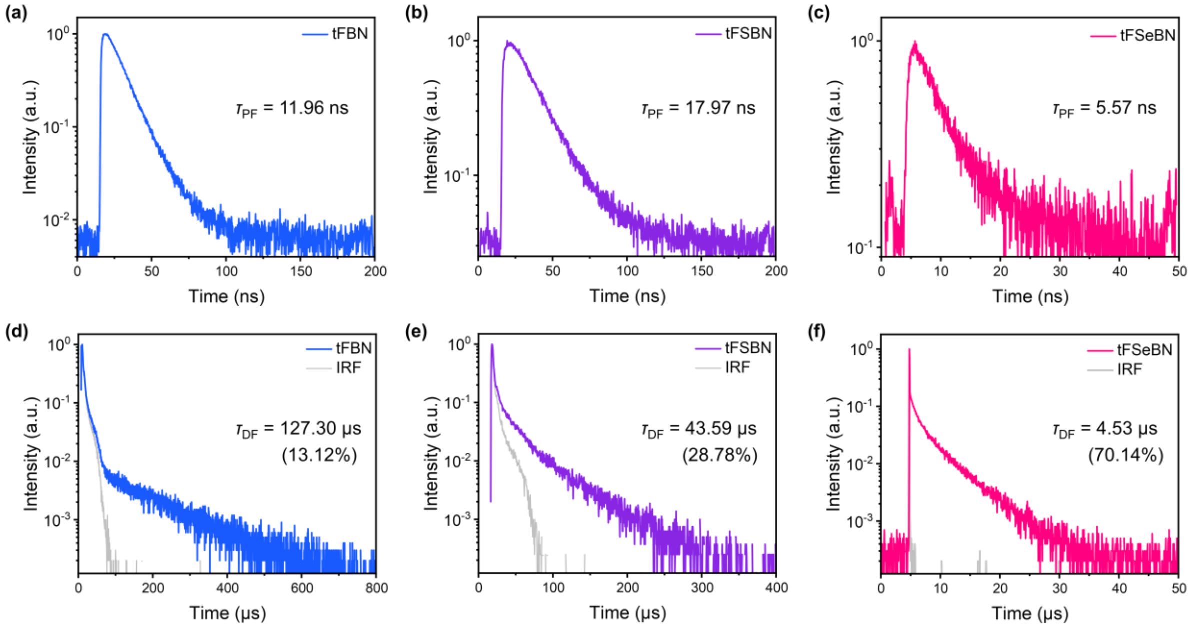

Fig. S19 Transient PL decay curves of **tFBN**, **tFSBN** and **tFSeBN** with 1 wt% doping concentration in DMIC-TRZ deposited films.

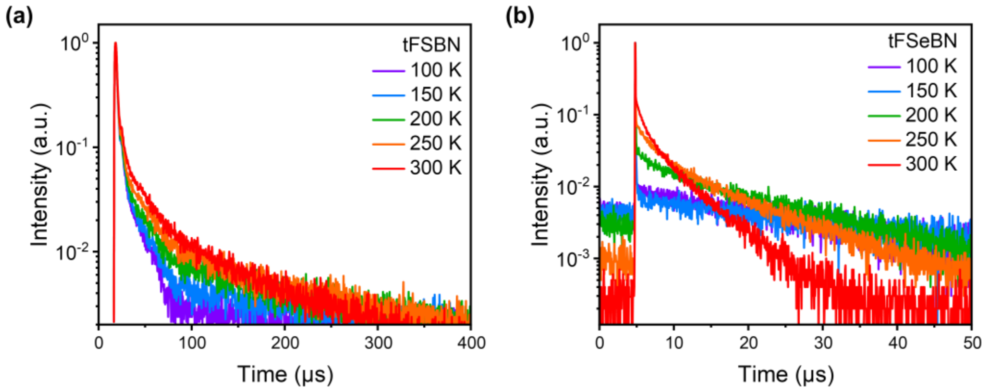

Fig. S20 Variable-temperature transient PL decay curves of **tFSBN** and **tFSeBN** with 1 wt% doping concentration in DMIC-TRZ deposited film.

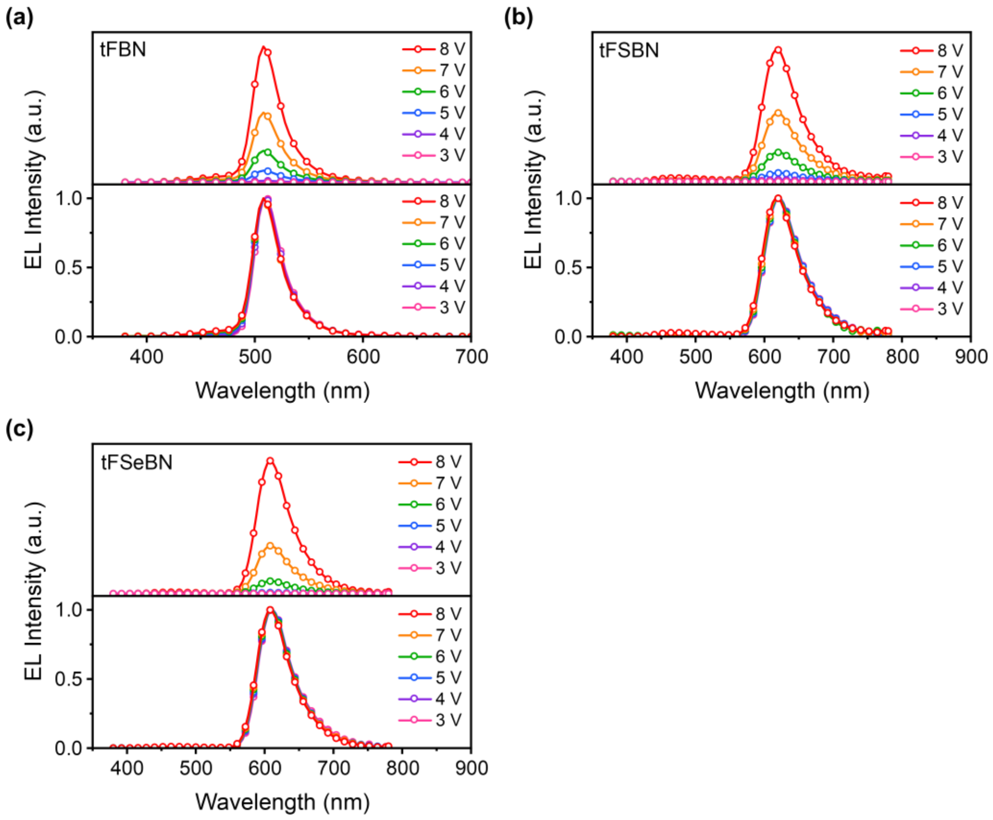

Fig. S21 Original (top) and normalized (bottom) EL spectra operated at different voltages of the non-sensitized OLEDs based on **tFBN**, **tFSBN** and **tFSeBN** at the dopant concentration of 1 wt%.

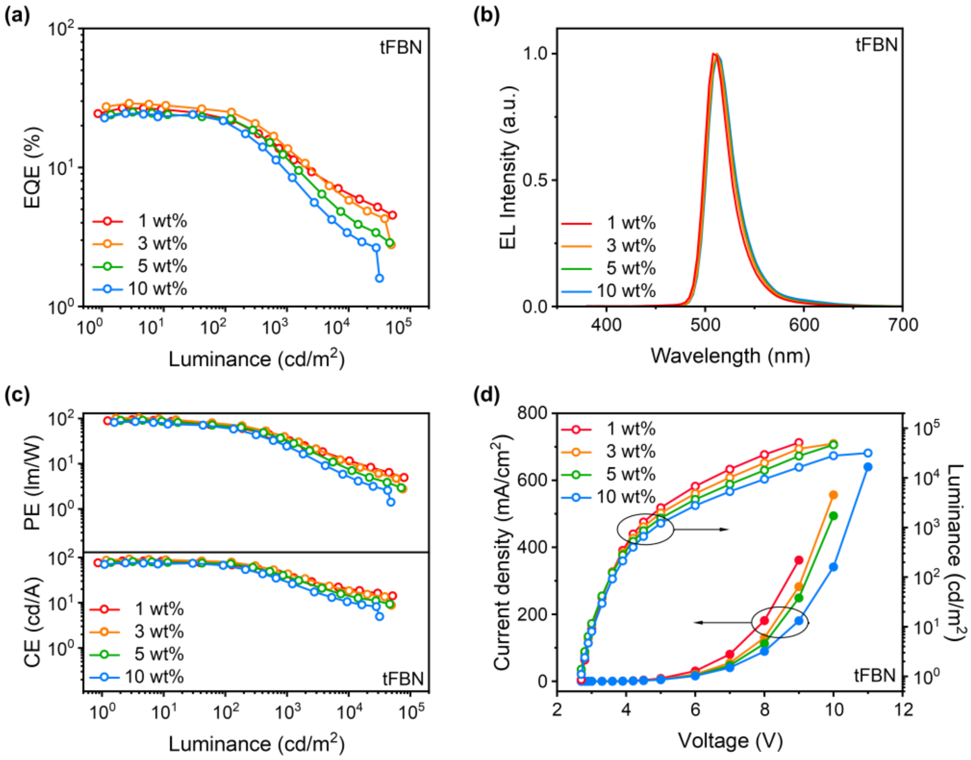

Fig. S22 Electroluminescence performance of the non-sensitized devices based on **tFBN** (DMIC-TRZ: x wt% **tFBN** (x = 1, 3, 5, 10) as EML). (a) External quantum efficiency versus luminance (EQE−*L*) curves. (b) Electroluminescence spectrum. (c) Current efficiency versus luminance (CE−*L*) and power efficiency versus luminance (PE−*L*) curves. (d) Current density and luminance versus voltage (*J*−*V*−*L*) characteristics.

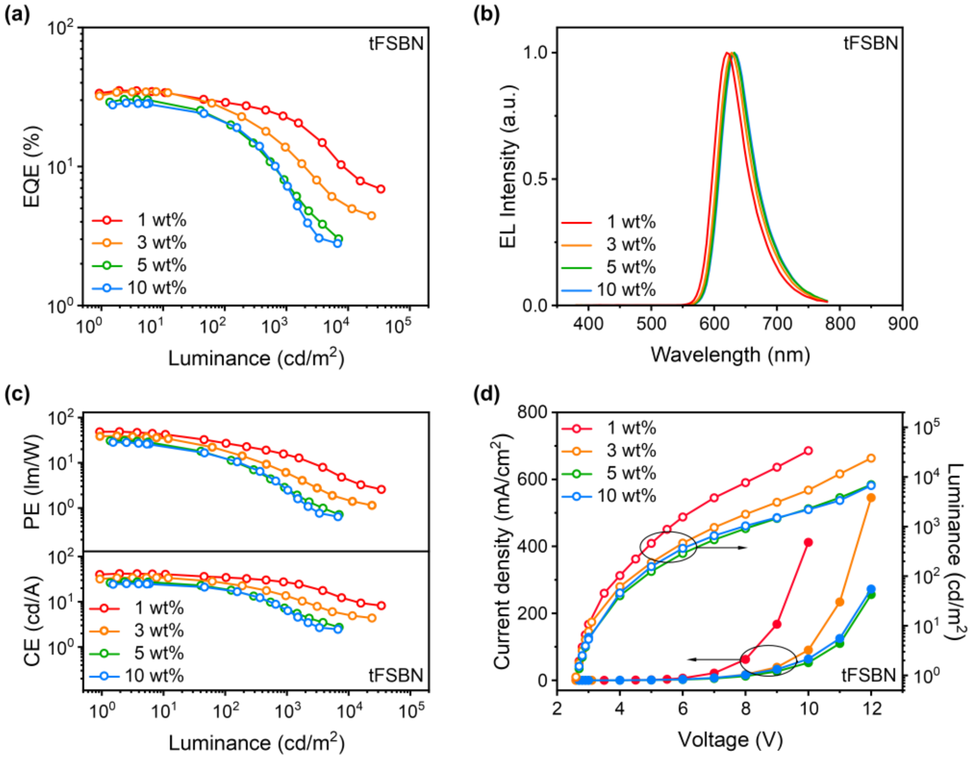

Fig. S23 Electroluminescence performance of the non-sensitized devices based on **tFSBN** (DMIC-TRZ: x wt% **tFSBN** (x = 1, 3, 5, 10) as EML). (a) External quantum efficiency versus luminance (EQE−*L*) curves. (b) Electroluminescence spectrum. (c) Current efficiency versus luminance (CE−*L*) and power efficiency versus luminance (PE−*L*) curves. (d) Current density and luminance versus voltage (*J*−*V*−*L*) characteristics.

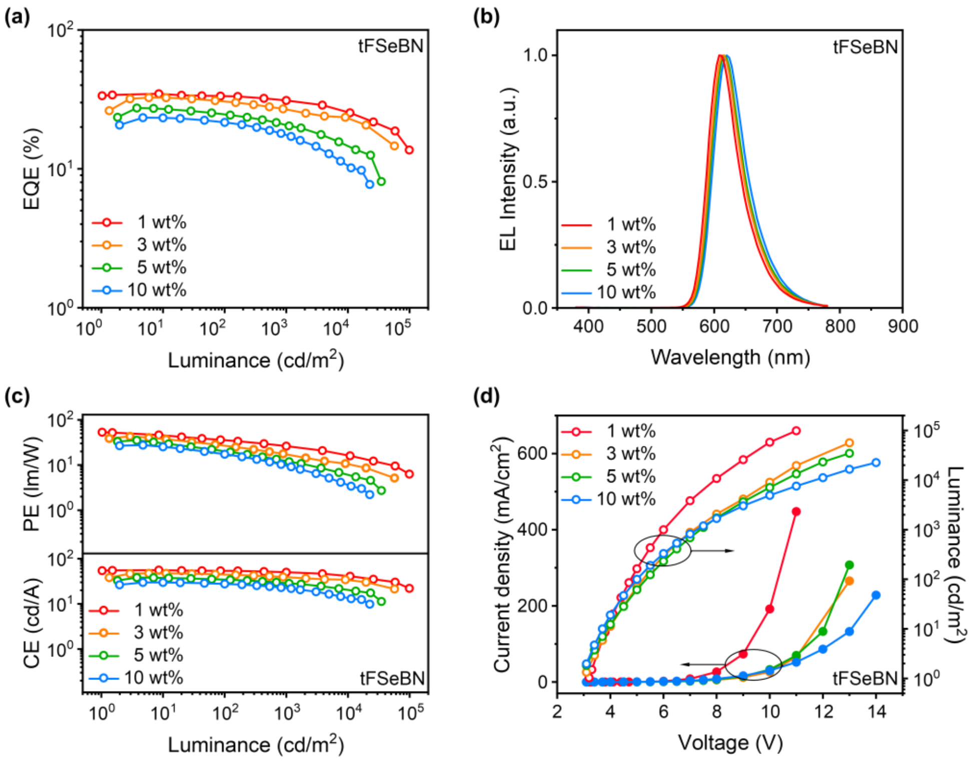

Fig. S24 Electroluminescence performance of the non-sensitized devices based on **tFSeBN** (DMIC-TRZ: x wt% **tFSeBN** (x = 1, 3, 5, 10) as EML). (a) External quantum efficiency versus luminance (EQE−*L*) curves. (b) Electroluminescence spectrum. (c) Current efficiency versus luminance (CE−*L*) and power efficiency versus luminance (PE−*L*) curves. (d) Current density and luminance versus voltage (*J*−*V*−*L*) characteristics.

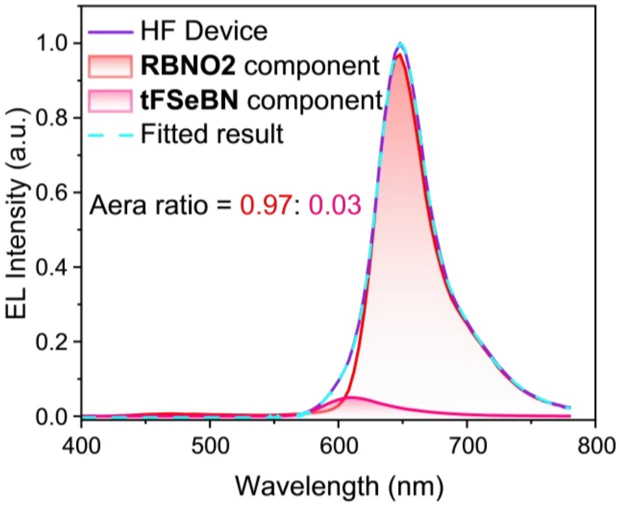

Fig. S25 Electroluminescence (EL) spectrum of the HF device and the corresponding fitting analysis.

Table S1 Summary EL performance of representative Se-containing MR-TADF OLEDs

Emitter	λ_{EL}[a)] [nm]	EQE_{max}[b)] [%]	$EQE_{1,000}$[c)] [%]	Roll-off ratio at 1,000 cd m^{-2} [%] [d)]	$EQE_{10,000}$[e)] [%]	Roll-off ratio at 10,000 cd m^{-2} [%] [f)]	Ref.
tFSeBN	608	34.7	31.0	**10.7**	25.6	**26.2**	this work
CzBSe	481	23.9	20.0	**16.0**	-	**-**	[1]
BNSSe	515	35.7	32.0	**10.4**	18.9	**47.1**	[2]
BNSeSe	512	36.8	34.0	**7.6**	21.9	**40.5**	[2]
BN-Se	506	32.6	32.2	**1.3**	24.0	**26.4**	[3]
BN-STO	517	40.1	28.1	**29.9**	-	**-**	[4]
mPXSe-BN	497	33.1	22.2	**32.9**	13.5	**59.2**	[5]
pPXSe-BN	500	31.7	26.7	**15.8**	18.8	**40.7**	[5]
PSeZBN1	475	25.4	14.7	**42.1**	6.4	**74.8**	[6]
PSeZBN2	507	27.2	26.5	**2.3**	20.4	**25.0**	[6]
Se-SFBN	488	35.6	22.1	**37.9**	-	**-**	[7]
DBDSe	456	40.5	28.2	**30.4**			[8]
DBSeBN	480	31.3	23.3	**25.6**	8.6	**72.5**	[9]
DBSe-BN	517	38.8	33.7	**13.1**	25.5	**34.3**	[10]

[a)] EL peak wavelength. [b)] Maximum external quantum efficiency. [c)] External quantum efficiency at 1,000 cd m^{-2}. [d)] Efficiency roll-off ratio at 1,000 cd m^{-2}. [e)] External quantum efficiency at 10,000 cd m^{-2}. [f)] Efficiency roll-off ratio at 10,000 cd m^{-2}.

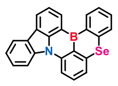
CzBSe

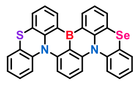
BNSSe

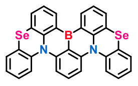
BNSeSe

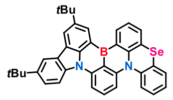
BN-Se

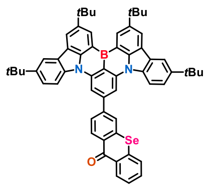
BN-STO

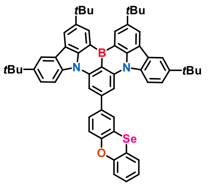
mPXSe-BN

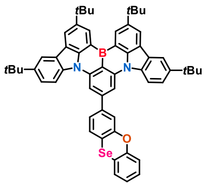
pPXSe-BN

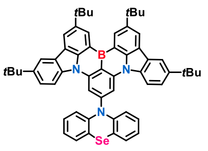
PSeZBN1

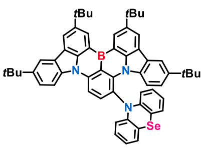
PSeZBN2

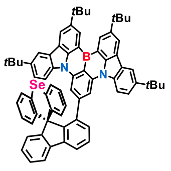
Se-SFBN

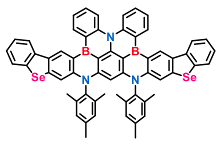
DBDSe

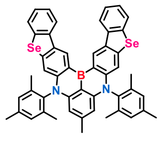
DBSeBN

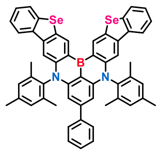
DBSe-BN

Table S2 Crystal data and structure refinement for **tFBN**.

Identification code	tFBN
Empirical formula	$C_{67}H_{71}BN_2$
Formula weight	915.06
Temperature/K	273.15
Crystal system	triclinic
Space group	P-1
a/Å	14.9623(7)
b/Å	17.4758(8)
c/Å	23.1124(10)
α/°	100.634(2)
β/°	100.859(2)
γ/°	97.062(2)
Volume/Å^3	5754.9(5)
Z	4
ρcalcg/cm^3	1.056
μ/mm^{-1}	0.449
F(000)	1968.0
Crystal size/mm^3	0.22 × 0.2 × 0.18
Radiation	CuKα (λ = 1.54178)
2Θ range for data collection/°	5.216 to 133.322
Index ranges	-17 ≤ h ≤ 16, -20 ≤ k ≤ 20, -27 ≤ l ≤ 27
Reflections collected	62730
Independent reflections	20105 [R_{int} = 0.0656, R_{sigma} = 0.0555]
Data/restraints/parameters	20105/986/1643
Goodness-of-fit on F^2	1.060
Final R indexes [I>=2σ (I)]	R_1 = 0.0772, wR_2 = 0.2019
Final R indexes [all data]	R_1 = 0.1062, wR_2 = 0.2264
Largest diff. peak/hole / e Å^{-3}	0.66/-0.41

Table S3 Crystal data and structure refinement for **tFSBN**.

Identification code	tFSBN
Empirical formula	$C_{67}H_{69}BN_2S$
Formula weight	945.11

Temperature/K	273.15
Crystal system	monoclinic
Space group	C2/c
a/Å	28.282
b/Å	36.793
c/Å	12.090
α/°	90
β/°	106.92
γ/°	90
Volume/Å^3	12036.5
Z	8
ρ_{calc}g/cm^3	1.043
μ/mm^{-1}	0.759
F(000)	4048.0
Crystal size/mm^3	0.22 × 0.2 × 0.18
Radiation	CuKα (λ = 1.54178)
2Θ range for data collection/°	4.804 to 133.674
Index ranges	$-33 \leq h \leq 25$, $-26 \leq k \leq 43$, $-14 \leq l \leq 14$
Reflections collected	20622
Independent reflections	10645 [R_{int} = 0.0587, R_{sigma} = 0.0642]
Data/restraints/parameters	10645/538/808
Goodness-of-fit on F^2	1.102
Final R indexes [I>=2σ (I)]	R_1 = 0.0818, wR_2 = 0.2432
Final R indexes [all data]	R_1 = 0.1063, wR_2 = 0.2664
Largest diff. peak/hole / e Å^{-3}	0.31/-0.32

Table S4 Crystal data and structure refinement for **FSeBN**.

Identification code	FSeBN
Empirical formula	$C_{59}H_{53}BN_2Se$
Formula weight	879.80
Temperature/K	273.15
Crystal system	triclinic
Space group	P-1
a/Å	16.4027(9)
b/Å	16.6738(8)

c/Å	19.7705(10)
α/°	76.404(2)
β/°	69.975(2)
γ/°	86.807(3)
Volume/$Å^3$	4936.1(4)
Z	4
ρcalcg/cm^3	1.184
μ/mm^{-1}	1.315
F(000)	1840.0
Crystal size/mm^3	0.12 × 0.1 × 0.08
Radiation	CuKα (λ = 1.54178)
2Θ range for data collection/°	4.888 to 134.796
Index ranges	-19 ≤ h ≤ 19, -19 ≤ k ≤ 19, -20 ≤ l ≤ 23
Reflections collected	52552
Independent reflections	17276 [R_{int} = 0.0986, R_{sigma} = 0.1180]
Data/restraints/parameters	17276/360/1282
Goodness-of-fit on F^2	1.072
Final R indexes [I>=2σ (I)]	R_1 = 0.0787, wR_2 = 0.2005
Final R indexes [all data]	R_1 = 0.1493, wR_2 = 0.2389
Largest diff. peak/hole / e $Å^{-3}$	0.37/-0.91

Table S5 Comparison of experimental and calculated excited-state energies for **tFBN**, **tFSBN**, and **tFSeBN**.

	Photophysical Measurements			TD-DFT Calculations			SCS-CC2 Calculations		
	S_1 [eV]	T_1 [eV]	ΔE_{ST} [eV]	S_1 [eV]	T_1 [eV]	ΔE_{ST} [eV]	S_1 [eV]	T_1 [eV]	ΔE_{ST}[eV]
tFBN	2.53	2.41	0.12	2.82	2.43	0.39	3.01	2.91	0.10
tFSBN	2.10	2.04	0.06	2.24	1.92	0.32	2.49	2.39	0.10
tFSeBN	2.15	2.07	0.08	2.28	1.97	0.31	2.58	2.49	0.09

Table S6 Summary performance in efficiency roll-off of several representative red MR-TADF OLEDs

	Non-sensitized device				Sensitized device				
Emitter	λ_{EL} [a)] [nm]	EQE_{max} [b)] [%]	$EQE_{1,000}$ [c)] [%]	Roll-off ratio[d)] [%]	λ_{EL} [a)] [nm]	EQE_{max} [b)] [%]	$EQE_{1,000}$ [c)] [%]	Roll-off ratio[d)] [%]	Ref.
tFSeBN	**608**	**34.7**	**31.0**	**10.7**	**-**	**-**	**-**	**-**	**This work**
tFSBN	620	35.2	22.5	**36.1**	-	-	-	-	
BN-R2	601	21.1	5.5	**73.9**	601	20.4	9.9	**51.5**	[11]
DB3	606	17.8	5.0	**71.9**	606	37.0	33.4	**9.7**	[12]
BNO1	609	14.9	3.3	**77.9**	610	35.6	31.1	**12.6**	[13]
BN-R1	610	20.7	6.3	**69.6**	611	19.0	9.1	**52.1**	[11]
3DPA-DiKTa	613	16.7	1.9	**88.6**	615	17.9	6.0	**66.5**	[14]
BNO3	616	15.1	4.5	**70.2**	625	36.1	32.1	**11.1**	[13]
BN-R	617	22.0	7.1	**67.7**	618	20.1	13.3	**33.8**	[15]
PTZBNO	617	13.6	6.3	**53.7**	618	34.5	29.7	**13.9**	[16]
BNTPA	617	35.2	24.6	**30.1**	619	43.3	37.7	**12.9**	[17]
BNCZ-DPAB	623	31.4	10.9	**65.3**	624	37.3	15.1	**59.5**	[18]
BNO2	623	12.0	4.4	**63.3**	618	34.4	29.8	**13.4**	[13]
FSBN	624	37.5	21.3	**43.2**	624	38.5	32.3	**16.1**	[19]
Δ-DABNA-TB	624	19.2	1.7	**91.2**	624	23.3	16.3	**30.0**	[20]
PXZBNO	630	11.8	3.9	**66.9**	632	28.1	23.8	**15.3**	[16]
RBNO1	632	17.2	3.0	**82.6**	632	33.1	22.5	**32.0**	[21]
RBNO2	645	15.1	3.4	**77.5**	645	34.7	28.8	**17.0**	[21]
2S-BN	676	29.3	16.7	**43.0**	680	28.2	20.9	**25.9**	[22]

[a)] EL peak wavelength. [b)] Maximum external quantum efficiency. [c)] External quantum efficiency at 1000 cd m^{-2}.
[d)] Efficiency roll-off ratio 1,000 cd m^{-2}. [e)] Commission Internationale de L'Eclairage coordinates.

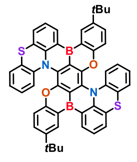

PTZBNO

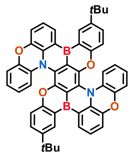

PXZBNO

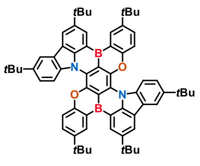

BNO1

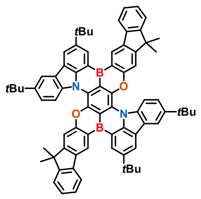

BNO2

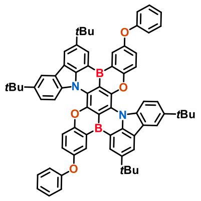

BNO3

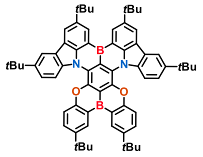

DB3

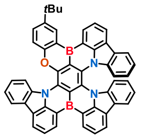

RBNO1

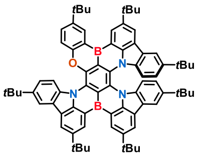

RBNO2

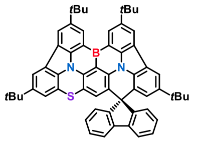

FSBN

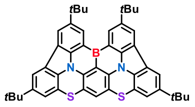

2S-BN

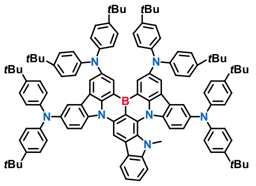

BNTPA

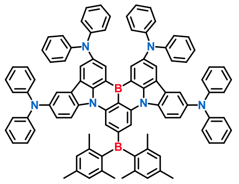

BNCZ-DPAB

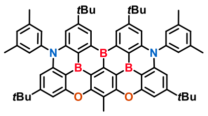

Δ-DABNA-TB

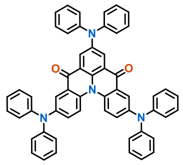

3DPA-DiKTa

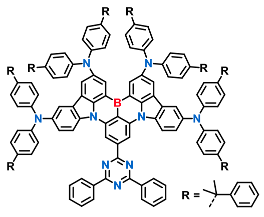

BN-R

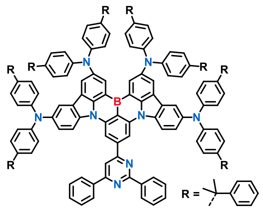

BN-R1

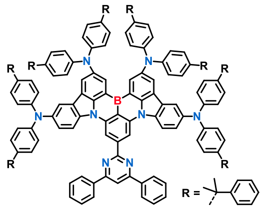

BN-R2

Table S7 The EL data of the non-sensitized devices based on **tFBN** (DMIC-TRZ: x wt% **tFBN** (x = 1, 3, 5, 10) as EML).

x wt%	λ_{EL} [a] [nm]	FWHM [b] [nm/eV]	V_{on} [c] [V]	L_{max} [d] [cd/m^2]	CE_{max} [e] [cd/A]	PE_{max} [f] [lm/W]	$EQE_{max/100/1,000/10,000}$ [g] [%]	CIE [h] (x, y)
1	508	29/0.14	2.7	51440	83.7	93.9	26.7/22.5/12.4/6.5	(0.14, 0.70)
3	512	30/0.14	2.7	49050	91.7	102.9	28.9/25.3/13.8/5.8	(0.15, 0.71)
5	512	30/0.14	2.7	46260	81.4	91.3	25.0/22.4/11.6/4.4	(0.16, 0.71)
10	512	30/0.14	2.7	31670	76.0	85.3	24.5/21.2/9.3/3.3	(0.17, 0.71)

[a] EL peak wavelength. [b] FWHM of EL spectra. [c] Turn-on voltage at the luminance of 1 cd m^{-2}. [d] Maximum luminance. [e] Maximum current efficiency. [f] Maximum power efficiency. [g] Maximum external quantum efficiency, and values at 100, 1,000 and 10,000 cd m^{-2}, respectively. [h] Commission Internationale de l'Éclairage coordinates (value taken at 100 cd m^{-2}).

Table S8 The EL data of the non-sensitized devices based on **tFSBN** (DMIC-TRZ:x wt% **tFSBN** (x = 1, 3, 5, 10) as EML).

x wt%	λ_{EL}[a)] [nm]	FWHM[b)] [nm/eV]	V_{on}[c)] [V]	L_{max}[d)] [cd/m^2]	CE_{max}[e)] [cd/A]	PE_{max}[f)] [lm/W]	$EQE_{max/100/1,000/10,000}$[g)] [%]	CIE[h)] (x, y)
1	620	59/0.19	2.6	33820	41.9	48.7	35.2/28.9/22.5/9.3	(0.67, 0.33)
3	628	60/0.19	2.6	23890	34.1	39.3	34.5/25.9/13.5/5.2	(0.67, 0.32)
5	632	60/0.19	2.7	6998	27.6	30.9	30.4/20.9/7.6/-	(0.68, 0.32)
10	632	61/0.19	2.7	6708	25.2	28.4	28.6/20.7/7.4/-	(0.68, 0.32)

a) EL peak wavelength. b) FWHM of EL spectra. c) Turn-on voltage at the luminance of 1 cd m^{-2}. d) Maximum luminance. e) Maximum current efficiency. f) Maximum power efficiency. g) Maximum external quantum efficiency, and values at 100, 1,000 and 10,000 cd m^{-2}, respectively. h) Commission Internationale de l'Éclairage coordinates (value taken at 100 cd m^{-2}).

Table S9 The EL data of the non-sensitized devices based on **tFSeBN** (DMIC-TRZ:x wt% **tFSeBN** (x = 1, 3, 5, 10) as EML).

x wt%	λ_{EL}[a)] [nm]	FWHM[b)] [nm/eV]	V_{on}[c)] [V]	L_{max}[d)] [cd/m^2]	CE_{max}[e)] [cd/A]	PE_{max}[f)] [lm/W]	$EQE_{max/100/1,000/10,000}$[g)] [%]	CIE[h)] (x, y)
1	608	57/0.19	3.1	98710	55.9	53.2	34.7/33.4/31.0/25.6	(0.64, 0.36)
3	612	58/0.19	3.1	56640	47.5	43.0	32.6/30.6/26.7/23.1	(0.65, 0.35)
5	616	58/0.19	3.1	34730	38.2	35.3	27.4/24.7/20.6/14.6	(0.65, 0.35)
10	620	60/0.19	3.1	22530	29.8	27.6	23.3/21.6/17.5/10.5	(0.66, 0.34)

a) EL peak wavelength. b) FWHM of EL spectra. c) Turn-on voltage at the luminance of 1 cd m^{-2}. d) Maximum luminance. e) Maximum current efficiency. f) Maximum power efficiency. g) Maximum external quantum efficiency, and values at 100, 1,000 and 10,000 cd m^{-2}, respectively. h) Commission Internationale de l'Éclairage coordinates (value taken at 100 cd m^{-2}).

Table S10. Simulation Molecular Cartesian coordinates.

Ground state geometry of **tFBN** in gas phase			
atom	x	y	z

B	2.468500	-1.805300	-1.575000	C	0.749500	0.302700	6.157000
C	2.501500	-6.837300	-3.084000	H	0.274500	0.888700	6.700000
C	1.221500	-7.768300	-3.211000	C	2.057500	-0.023300	6.451000
H	0.456500	-7.233300	-3.431000	H	2.456500	0.369700	7.192000
H	1.070500	-8.219300	-2.377000	C	2.803500	-0.911300	5.689000
H	1.367500	-8.419300	-3.903000	C	2.193500	-1.439300	4.544000
C	2.623500	-6.218300	-4.491000	H	2.667500	-2.025300	3.999000
H	2.591500	-6.915300	-5.152000	C	4.237500	-1.262300	6.111000
H	3.456500	-5.747300	-4.564000	C	4.859500	-2.357300	5.247000
H	1.896500	-5.607300	-4.637000	H	4.323500	-3.151300	5.303000
C	3.595500	-7.643300	-2.575000	H	5.747500	-2.546300	5.560000
H	3.309500	-8.109300	-1.785000	H	4.899500	-2.058300	4.334000
H	4.339500	-7.075300	-2.359000	C	4.287500	-1.649300	7.578000
H	3.861500	-8.280300	-3.242000	H	3.828500	-0.986300	8.101000
C	2.171500	-5.739300	-2.061000	H	5.200500	-1.705300	7.866000
C	1.553500	-6.050300	-0.839000	H	3.861500	-2.501300	7.698000
H	1.336500	-6.933300	-0.645000	C	5.119500	-0.009300	5.926000
C	1.263500	-5.059300	0.081000	H	5.164500	0.216000	4.994400
C	1.639500	-3.746300	-0.270000	H	4.740600	0.724100	6.415000
C	2.240500	-3.337300	-1.452000	H	6.152400	-0.218200	6.311100
C	2.483500	-4.406300	-2.334000	C	-1.210500	-0.146300	4.504000
H	2.878500	-4.212300	-3.153000	C	-1.285500	-0.898300	3.331000
C	0.579500	-4.957300	1.376000	C	-2.462500	-0.982300	2.625000
C	0.586500	-3.604300	1.703000	H	-2.494500	-1.482300	1.841000
C	-0.007500	-3.028300	2.806000	C	-3.603500	-0.330300	3.071000
C	-0.644500	-3.929300	3.650000	C	-3.507500	0.413700	4.246000
H	-1.065500	-3.604300	4.413000	H	-4.260500	0.861700	4.558000
C	-0.679500	-5.313300	3.397000	C	-2.332500	0.509700	4.962000
C	-0.075500	-5.822300	2.261000	H	-2.296500	1.010700	5.744000
H	-0.104500	-6.735300	2.087000	C	-4.918500	-0.364300	2.284000
C	-1.431500	-6.224300	4.377000	C	-6.091500	-0.626300	3.165000
C	-2.884500	-5.819300	4.520000	H	-6.052500	-1.527300	3.493000
H	-2.945500	-5.026300	5.057000	H	-6.901500	-0.503300	2.664000
H	-3.374500	-6.528300	4.939000	H	-6.078500	-0.014300	3.906000
H	-3.254500	-5.645300	3.651000	C	-5.136500	1.058700	1.672000
C	-0.705500	-6.123300	5.767000	H	-5.182500	1.707700	2.380000
H	0.168500	-6.512300	5.700000	H	-5.958500	1.071700	1.176000
H	-1.215500	-6.595300	6.429000	H	-4.406500	1.272700	1.089000
H	-0.627500	-5.200300	6.021000	C	-4.912500	-1.306300	1.120000
C	0.070500	-1.508300	2.989000	H	-4.138500	-1.139300	0.579000
C	0.898500	-1.100300	4.216000	H	-5.704500	-1.172300	0.597000
C	0.155500	-0.256300	5.039000	H	-4.889500	-2.210300	1.442000

C	0.656500	-0.809300	1.732000
C	0.628500	0.560700	1.580000
H	0.175500	1.069700	2.213000
C	1.245500	1.212700	0.527000
H	1.189500	2.137700	0.464000
C	1.947500	0.496700	-0.439000
C	1.900500	-0.921300	-0.429000
C	1.269500	-1.511300	0.686000
C	3.162500	-1.003300	-2.689000
C	3.259500	0.378700	-2.499000
C	3.926500	1.232700	-3.365000
C	4.498500	0.708700	-4.523000
H	4.946500	1.270700	-5.112000
C	4.404500	-0.654300	-4.802000
C	3.767500	-1.465300	-3.877000
H	3.735500	-2.378300	-4.052000
C	5.025500	-1.226300	-6.089000
C	6.524500	-1.259300	-5.965000
H	6.859500	-0.364300	-5.877000
H	6.901500	-1.665300	-6.751000
H	6.771500	-1.771300	-5.192000
C	4.598500	-0.380300	-7.292000
H	3.640500	-0.320300	-7.318000
H	4.913500	-0.791300	-8.101000
H	4.973500	0.499700	-7.214000
C	4.544500	-2.655300	-6.394000
H	4.904500	-3.260300	-5.742000
H	4.840500	-2.914300	-7.267000
H	3.584500	-2.685300	-6.360000
C	3.818500	2.567700	-2.814000
C	3.055500	2.469700	-1.640000
C	2.799500	3.610700	-0.906000
H	2.317500	3.565700	-0.113000
C	3.275500	4.820700	-1.376000
H	3.096500	5.584700	-0.878000
C	4.006500	4.962700	-2.550000
C	4.281500	3.793700	-3.257000
H	4.783500	3.837700	-4.038000
C	4.460500	6.319700	-3.095000
C	5.953500	6.275700	-3.430000
H	6.116900	5.584000	-4.076000
H	6.540500	6.063500	-2.497600
H	6.271100	7.263900	-3.855800
C	3.662500	6.613700	-4.371000
H	3.788500	5.899700	-4.999000
H	3.970500	7.437700	-4.759000
H	2.730500	6.690700	-4.156000
C	4.237500	7.453700	-2.098000
H	3.301900	7.522200	-1.894700
H	4.727600	7.273600	-1.293400
H	4.595900	8.419300	-2.542800
N	2.697500	1.113700	-1.450000
N	1.242500	-2.888300	0.726000
C	-1.059500	-7.689300	4.085000
H	-1.325500	-7.914300	3.189000
H	-1.522500	-8.263300	4.704000
H	-0.116500	-7.808300	4.183000

Ground state geometry of **tFSBN** in gas phase

atom	x	y	z
B	1.050500	-2.034500	-2.039500
C	-7.135500	-0.132500	-2.677500
C	-4.876500	-1.141500	-3.254500
H	-5.303500	-1.626500	-3.924500
C	-5.599500	-0.246500	-2.514500
C	-4.921500	0.506500	-1.531500
H	-5.404500	1.125500	-1.035500
C	-3.573500	0.364500	-1.274500
C	-2.912500	-0.570500	-2.040500
C	-3.532500	-1.339500	-3.030500
C	-2.466500	-2.202500	-3.564500
C	-1.303500	-1.890500	-2.868500
C	-0.046500	-2.511500	-2.999500
C	-0.064500	-3.515500	-3.996500
H	0.728500	-3.970500	-4.166500
C	-1.191500	-3.866500	-4.743500
C	-2.389500	-3.213500	-4.507500
H	-3.148500	-3.458500	-4.985500
C	-1.051500	-4.966500	-5.804500
C	-2.383500	-5.272500	-6.522500
H	-3.039500	-5.533500	-5.874500
H	-2.253500	-5.982500	-7.155500
H	-2.682500	-4.485500	-6.982500
C	-0.543500	-6.239500	-5.143500

H	0.322500	-6.067500	-4.765500	H	6.108500	-4.253500	-4.213500
H	-0.470500	-6.935500	-5.801500	H	6.900500	-5.522500	-3.749500
H	-1.147500	-6.516500	-4.450500	H	6.943500	-4.216500	-2.887500
C	-0.034500	-4.498500	-6.854500	C	1.641500	1.577500	3.048500
H	-0.305500	-3.646500	-7.199500	C	1.525500	2.927500	3.336500
H	0.005500	-5.136500	-7.571500	C	0.939500	3.308500	4.531500
H	0.832500	-4.419500	-6.449500	H	0.887500	4.208500	4.758500
C	-1.067500	0.641500	-0.148500	C	0.441500	2.366500	5.370500
C	-0.667500	-0.366500	-1.053500	H	0.034500	2.645500	6.159500
C	0.627500	-0.893500	-1.078500	C	0.514500	0.996500	5.102500
C	1.526500	-0.303500	-0.171500	C	1.141500	0.625500	3.919500
C	1.205500	0.712500	0.733500	H	1.225500	-0.276500	3.710500
C	-0.110500	1.150500	0.715500	C	-0.162500	-0.057500	5.990500
H	-0.360500	1.817500	1.312500	C	2.513500	2.801500	1.231500
C	2.252500	1.355500	1.670500	C	2.081500	3.689500	2.202500
C	3.569500	0.574500	1.686500	C	2.240500	5.049500	1.991500
C	3.756500	-0.419500	0.740500	H	1.963500	5.669500	2.625500
C	4.897500	-1.215500	0.587500	C	2.825500	5.452500	0.809500
C	5.953500	-0.950500	1.441500	H	2.925500	6.364500	0.660500
H	6.738500	-1.446500	1.376500	C	3.276500	4.579500	-0.170500
C	5.842500	0.066500	2.403500	C	3.103500	3.224500	0.069500
C	4.636500	0.786500	2.511500	H	3.390500	2.600500	-0.558500
H	4.562500	1.434500	3.173500	C	3.982500	5.083500	-1.431500
C	7.013500	0.364500	3.327500	C	4.127500	4.039500	-2.499500
C	4.581500	-2.149500	-0.471500	H	4.585500	3.263500	-2.165500
C	3.272500	-1.869500	-0.892500	H	4.610500	4.396500	-3.250500
C	2.508500	-2.557500	-1.835500	H	3.241500	3.793500	-2.774500
C	3.202500	-3.659500	-2.386500	C	5.443500	5.441500	-1.004500
H	2.764500	-4.179500	-3.021500	H	5.438500	6.063500	-0.274500
C	4.487500	-4.009500	-2.041500	H	5.915500	5.820500	-1.747500
C	5.194500	-3.247500	-1.087500	H	5.877500	4.628500	-0.729500
H	6.068500	-3.473500	-0.866500	C	3.411500	6.416500	-1.866500
C	5.191500	-5.223500	-2.683500	H	2.486500	6.313500	-2.098500
C	4.235500	-6.043500	-3.590500	H	3.901500	6.732500	-2.631500
H	3.424500	-6.207500	-3.106500	H	3.496500	7.049500	-1.150500
H	4.655500	-6.880500	-3.807500	N	-1.582500	-0.890500	-1.936500
H	4.035500	-5.567500	-4.398500	N	2.798500	-0.791500	-0.172500
C	5.639500	-6.182500	-1.537500	S	-2.705500	1.303500	-0.060500
H	6.428500	-5.828500	-1.119500	C	7.372500	1.740500	2.721500
H	5.833500	-7.049500	-1.905500	H	6.997500	1.824500	1.843500
H	4.942500	-6.262500	-0.883500	H	8.321500	1.865500	2.679500
C	6.394500	-4.764500	-3.453500	H	6.987500	2.405500	3.299500

C	8.023500	-0.795500	3.149500
H	7.580500	-1.630500	3.311500
H	8.748500	-0.695500	3.775500
H	8.362500	-0.772500	2.257500
C	6.436500	0.128500	4.729500
H	5.961500	0.904500	5.034500
H	7.146500	-0.077500	5.343500
H	5.832500	-0.614500	4.678500
C	-7.709500	-0.725500	-3.965500
H	-8.662500	-0.625500	-3.966500
H	-7.484500	-1.659500	-3.989500
H	-7.339500	-0.287500	-4.735500
C	-7.796500	-0.739500	-1.429500
H	-7.556500	-0.198500	-0.674500
H	-7.517500	-1.644500	-1.275500
H	-8.748500	-0.714500	-1.554500
C	-7.292500	1.412500	-2.668500
H	-6.645500	1.751500	-3.298500
H	-7.124500	1.791500	-1.806500
H	-8.175500	1.655500	-2.962500
C	-1.555500	0.319500	6.503500
H	-1.345500	1.199500	6.827500
H	-1.741500	-0.257500	7.253500
H	-2.328500	0.363500	5.938500
C	-0.248500	-1.468500	5.358500
H	-0.840500	-1.611500	4.617500
H	-0.465500	-2.086500	6.063500
H	0.656500	-1.608500	5.080500
C	1.032500	-0.313500	6.920500
H	1.870500	-0.246500	6.453500
H	0.980500	-1.162500	7.363500
H	0.976500	0.389500	7.571500

Ground state geometry of **tFSeBN** in gas phase

atom	x	y	z
Se	-3.859000	-1.357000	-2.393000
N	0.556000	1.443000	0.651000
N	-1.890000	-2.565000	-0.144000
C	-1.687000	-1.209000	-0.408000
C	2.176000	5.078000	-0.322000
C	-3.690000	-3.126000	-1.779000
C	-2.199000	3.735000	-0.350000
C	0.299000	3.525000	-0.576000
C	-2.765000	-3.450000	-0.790000
C	-1.094000	-3.297000	0.714000
C	-0.473000	0.797000	-0.018000
C	1.509000	0.855000	1.441000
C	-1.059000	3.043000	-1.111000
C	-0.034000	-2.808000	1.466000
C	-1.303000	3.562000	-2.523000
C	2.201000	2.987000	0.924000
C	2.821000	6.400000	-0.773000
C	-2.214000	0.815000	-1.600000
H	-2.727000	1.263000	-2.234000
C	0.965000	2.717000	0.315000
C	2.798000	4.218000	0.586000
H	3.610000	4.458000	0.969000
C	-4.392000	-4.191000	-2.319000
H	-4.989000	-4.022000	-3.011000
C	0.642000	-3.788000	2.215000
H	1.361000	-3.518000	2.741000
C	0.299000	-5.145000	2.213000
C	0.934000	4.716000	-0.896000
H	0.534000	5.292000	-1.507000
C	-2.480000	-0.537000	-1.357000
C	-0.780000	-5.566000	1.439000
H	-1.024000	-6.464000	1.435000
C	2.543000	1.794000	1.678000
C	-0.645000	-0.576000	0.293000
C	1.463000	-0.474000	1.864000
C	-1.493000	-4.655000	0.674000
C	-2.380000	4.429000	-2.567000
C	3.621000	0.043000	2.961000
C	3.611000	1.358000	2.459000
H	4.315000	1.934000	2.648000
C	-4.244000	-5.524000	-1.867000
C	-1.227000	1.512000	-0.936000
C	-2.961000	4.502000	-1.222000
C	-3.321000	-5.792000	-0.851000
H	-3.203000	-6.659000	-0.540000
C	2.589000	-0.837000	2.639000
H	2.644000	-1.712000	2.950000
C	-2.577000	-4.729000	-0.309000
C	-2.527000	3.635000	0.973000

H	-2.022000	3.101000	1.544000	H	5.451000	-2.196000	4.763000
C	1.170000	-6.166000	2.972000	H	4.656000	-2.488000	3.444000
C	-0.540000	3.326000	-3.655000	H	3.888000	-2.097000	4.752000
H	0.174000	2.731000	-3.637000	C	4.874000	0.542000	5.018000
C	-4.987000	-6.672000	-2.561000	H	4.174000	0.384000	5.647000
C	-4.375000	5.099000	0.611000	H	4.801000	1.440000	4.673000
H	-5.110000	5.559000	0.949000	H	5.728000	0.449000	5.457000
C	-2.701000	5.104000	-3.743000	C	6.087000	-0.412000	3.030000
H	-3.418000	5.693000	-3.772000	H	6.660000	0.270000	3.379000
C	-3.614000	4.335000	1.450000	H	5.881000	-0.227000	2.113000
C	4.782000	-0.467000	3.852000	H	6.533000	-1.267000	3.089000
C	4.068000	6.733000	0.056000	C	-3.985000	-7.489000	-3.411000
H	3.827000	6.794000	0.984000	H	-3.251000	-7.770000	-2.858000
H	4.434000	7.571000	-0.237000	H	-3.655000	-6.943000	-4.128000
H	4.722000	6.039000	-0.059000	H	-4.426000	-8.261000	-3.772000
C	3.240000	6.237000	-2.236000	C	-5.609000	-7.606000	-1.506000
H	3.884000	5.529000	-2.308000	H	-5.985000	-8.375000	-1.941000
H	3.630000	7.056000	-2.550000	H	-6.299000	-7.138000	-1.030000
H	2.470000	6.025000	-2.767000	H	-4.930000	-7.888000	-0.889000
C	-4.074000	5.199000	-0.726000	C	-6.095000	-6.224000	-3.530000
H	-4.597000	5.719000	-1.292000	H	-6.619000	-6.985000	-3.792000
C	-0.876000	4.008000	-4.824000	H	-5.698000	-5.827000	-4.309000
C	1.666000	-5.596000	4.315000	H	-6.660000	-5.582000	-3.096000
H	0.912000	-5.329000	4.846000	C	-0.044000	3.777000	-6.099000
H	2.229000	-4.837000	4.152000	C	1.133000	2.837000	-5.781000
H	2.164000	-6.270000	4.783000	H	1.732000	3.266000	-5.005000
C	1.823000	7.561000	-0.672000	H	0.756000	1.890000	-5.458000
H	1.073000	7.387000	-1.243000	H	1.729000	2.703000	-6.659000
H	2.252000	8.375000	-0.945000	C	-0.932000	3.138000	-7.183000
H	1.523000	7.646000	0.235000	H	-1.716000	3.817000	-7.447000
C	-1.932000	4.881000	-4.855000	H	-0.340000	2.923000	-8.048000
H	-2.131000	5.330000	-5.647000	H	-1.358000	2.232000	-6.806000
B	0.286000	-1.308000	1.282000	C	0.497000	5.126000	-6.609000
C	0.386000	-7.429000	3.267000	H	1.207000	4.952000	-7.390000
H	0.963000	-8.076000	3.679000	H	-0.311000	5.715000	-6.986000
H	0.038000	-7.788000	2.448000	H	0.973000	5.646000	-5.804000
H	-0.339000	-7.225000	3.861000	C	-3.976000	4.253000	2.944000
C	2.383000	-6.490000	2.092000	C	-4.643000	2.897000	3.237000
H	2.873000	-5.684000	1.913000	H	-5.184000	2.571000	2.373000
H	2.082000	-6.873000	1.264000	H	-3.892000	2.175000	3.481000
H	2.950000	-7.115000	2.547000	H	-5.318000	3.001000	4.061000
C	4.684000	-1.950000	4.241000	C	-2.697000	4.390000	3.791000

H	-2.176000	3.456000	3.804000	H	-5.873000	5.249000	2.773000
H	-2.068000	5.144000	3.367000	H	-5.136000	5.388000	4.352000
H	-2.960000	4.666000	4.791000	H	-4.522000	6.330000	3.015000
C	-4.951000	5.392000	3.298000				

References

1. Park, I. S., Min, H. & Yasuda, T. Ultrafast Triplet-Singlet Exciton Interconversion in Narrowband Blue Organoboron Emitters Doped with Heavy Chalcogens. *Angew. Chem. Int. Ed.* **61**, e202205684 (2022).
2. Hu, Y. X. *et al.* Efficient selenium-integrated TADF OLEDs with reduced roll-off. *Nat. Photon.* **16**, 803–810 (2022).
3. Cao, X. *et al.* Manipulating Exciton Dynamics toward Simultaneous High-Efficiency Narrowband Electroluminescence and Photon Upconversion by a Selenium-Incorporated Multiresonance Delayed Fluorescence Emitter. *J. Am. Chem. Soc.* **144**, 22976–22984 (2022).
4. Hu, Y. *et al.* Peripherally Heavy-Atom-Decorated Strategy Towards High-Performance Pure Green Electroluminescence with External Quantum Efficiency over 40. *Angew. Chem. Int. Ed.* **62**, e202302478 (2023).
5. Chen, Z. *et al.* Advancing Triplet Exciton Harvesting Through Heavy Atom Selenium Manipulation in Multiple Resonance Thermally Activated Delayed Fluorescent Emitters. *Adv. Funct. Mater.* **34**, 2404278 (2024).
6. Zou, Y. *et al.* Acceleration of reverse intersystem crossing in multi-resonance TADF emitter. *Chem* **10**, 1485–1501 (2024).
7. Zheng, Q. *et al.* Enhancing multi-resonance thermally activated delayed fluorescence emission via through-space heavy-atom effect. *Chem* **11**, 1–11 (2025).
8. Lin, H. *et al.* Deep-Blue Narrowband OLEDs Achieve External Quantum Efficiency Over 40% and Blue Index of 422 by Synergistic π-Extension and Heavy-Atom Effect. *Adv. Mater* **37**, e2502459 (2025).
9. Chen, Z. *et al.* Narrow-Band Dibenzoselenophene-Based Emitter with Rapid Triplet Conversion for Versatile OLED Applications with Superior Roll-Off Suppression. *Angew. Chem. Int. Ed.* **64**, e202507626 (2025).
10. Liu, J. *et al.* Increasing Aromaticity in Selenium-Embedded Hetero Ring Enables Stable Narrowband Multi-Resonance TADF Emitter for Long-Lifetime Pure-Green OLEDs. *Adv. Mater.* https://doi.org/10.1002/adma.202513987 (2025).

11. Cai, X. *et al.* Precisely Regulation of Peripheral Decoration of Multi-Resonance Molecules and Construction of Highly Efficient Solution-Processed Orange–Red OLEDs with External Quantum Efficiency Approaching 20%. *Adv. Opt. Mater.* **12**, 2302811 (2023).
12. Miao, J. *et al.* Modulation of Carbazole/Phenol Resonant Partners within Diboron-Based Multi-Resonance Emitters Enable High-Performance Narrowband Green to Red OLEDs. *Adv. Funct. Mater.* **34**, 2316323 (2024).
13. Zou, Y. *et al.* High-Performance Narrowband Pure-Red OLEDs with External Quantum Efficiencies up to 36.1% and Ultralow Efficiency Roll-Off. *Adv. Mater.* **34**, e2201442 (2022).
14. Wu, S. *et al.* Highly Efficient Green and Red Narrowband Emissive Organic Light-Emitting Diodes Employing Multi-Resonant Thermally Activated Delayed Fluorescence Emitters. *Angew. Chem. Int. Ed.* **61**, e202213697 (2022).
15. Cai, X. *et al.* Solution-Processable Pure-Red Multiple Resonance-induced Thermally Activated Delayed Fluorescence Emitter for Organic Light-Emitting Diode with External Quantum Efficiency over 20. *Angew. Chem. Int. Ed.* **62**, e202216473 (2023).
16. He, J. *et al.* Phenoxazine and phenothiazine embedded Multi-Resonance emitters for highly efficient Pure-Red OLEDs with improved color purity. *Chem. Eng. J.* **471**, 144565 (2023).
17. Ge, L. *et al.* Efficient and Stable Narrowband Pure-Red Light-Emitting Diodes with Electroluminescence Efficiencies Exceeding 43. *J. Am. Chem. Soc.* **146**, 32826–32836 (2024).
18. Wang, H. *et al.* Efficient solution-processable OLEDs near BT.2020 red standard enabled by a multiresonant emitter. *Sci. Bull. (Beijing)* **69**, 2983–2986 (2024).
19. Pu, Y. *et al.* Spiro-Carbon-Locking and Sulfur-Embedding Strategy for Constructing Deep-Red Organic Electroluminescent Emitter with High Efficiency. *Angew. Chem. Int. Ed.* **64**, e202420253 (2025).
20. Hayakawa, M. *et al.* "Core-Shell" Wave Function Modulation in Organic Narrowband Emitters. *J. Am. Chem. Soc.* **146**, 18331–18340 (2024).
21. Zou, Y. *et al.* Precisely regulating the double-boron-based multi-resonance framework

towards pure-red emitters: high-performance OLEDs with CIE coordinates fully satisfying the BT. 2020 standard. *Mater. Horiz.* **10**, 3712–3718 (2023).

22. Pu, Y. *et al.* Sulfur-locked multiple resonance emitters for high performance orange-red/deep-red OLEDs. *Nat. Commun.* **16**, 332 (2025).
